# Modeling and simulation of the main metabolism in *Escherichia coli *and its several single-gene knockout mutants with experimental verification

**DOI:** 10.1186/1475-2859-9-88

**Published:** 2010-11-19

**Authors:** Tuty Asmawaty Abdul Kadir, Ahmad A Mannan, Andrzej M Kierzek, Johnjoe McFadden, Kazuyuki Shimizu

**Affiliations:** 1 Dept. of Bioscience and Bioinformatics, Kyushu Institute of Technology, Iizuka, Fukuoka 820-8502, Japan; 2 Fac. Of Health and Medical Sciences, AW Building, University of Surrey, Guilford Surrey GU2 7TE, UK; 3 Institute for Advanced Biosciences, Keio University, Tsuruoka, Yamagata 997-0017, Japan

## Abstract

**Background:**

It is quite important to simulate the metabolic changes of a cell in response to the change in culture environment and/or specific gene knockouts particularly for the purpose of application in industry. If this could be done, the cell design can be made without conducting exhaustive experiments, and one can screen out the promising candidates, proceeded by experimental verification of a select few of particular interest. Although several models have so far been proposed, most of them focus on the specific metabolic pathways. It is preferred to model the whole of the main metabolic pathways in *Escherichia coli*, allowing for the estimation of energy generation and cell synthesis, based on intracellular fluxes and that may be used to characterize phenotypic growth.

**Results:**

In the present study, we considered the simulation of the main metabolic pathways such as glycolysis, TCA cycle, pentose phosphate (PP) pathway, and the anapleorotic pathways using enzymatic reaction models of *E. coli*. Once intracellular fluxes were computed by this model, the specific ATP production rate, the specific CO_2 _production rate, and the specific NADPH production rate could be estimated. The specific ATP production rate thus computed was used for the estimation of the specific growth rate. The CO_2 _production rate could be used to estimate cell yield, and the specific NADPH production rate could be used to determine the flux of the oxidative PP pathway. The batch and continuous cultivations were simulated where the changing patterns of extracellular and intra-cellular metabolite concentrations were compared with experimental data. Moreover, the effects of the knockout of such pathways as Ppc, Pck and Pyk on the metabolism were simulated. It was shown to be difficult for the cell to grow in Ppc mutant due to low concentration of OAA, while Pck mutant does not necessarily show this phenomenon. The slower growth rate of the Ppc mutant was properly estimated by taking into account the lower specific ATP production rate. In the case of Pyk mutant, the enzyme level regulation was made clear such that Pyk knockout caused PEP concentration to be up-regulated and activated Ppc, which caused the increase in MAL concentration and backed up reduced PYR through Mez, resulting in the phenotypic growth characteristics similar to the wild type.

**Conclusions:**

It was shown to be useful to simulate the main metabolism of *E. coli *for understanding metabolic changes inside the cell in response to specific pathway gene knockouts, considering the whole main metabolic pathways. The comparison of the simulation result with the experimental data indicates that the present model could simulate the effect of the specific gene knockouts to the changes in the metabolisms to some extent.

## Background

One of the most challenging goals of metabolic engineering is to design the cell metabolism based on the analysis of metabolic regulation. For this, it is strongly desired to develop a mathematical model which can describe the dynamic behaviour of the cell in response to the changes in the culture environment and/or specific genetic modifications. Although an attempt has been made to develop a platform for the whole cell model [1], the total cell model has not yet been developed. If such a model could be developed, it becomes possible to check the metabolism of a specific gene knockout on the metabolism and fermentation characteristics without conducting many exhaustive experiments, and allow for the screening out of the preferred candidates for performance improvement, followed by experimental verification only for the selected candidates.

Some of the mathematical models which can describe the dynamic behaviour of the intracellular metabolite concentrations of the central metabolic pathways have been developed for *Saccharomyces cerevisiae *[2-4]. The measurement of the intracellular metabolite concentrations for the pulse addition of glucose during continuous culture has been made, and the time profile was compared to the predicted dynamic simulation together with model parameter identification [3,5-7]. The kinetic equations for the glycolysis and the pentose phosphate (PP) pathway have also been developed for *E. coli *to simulate the transient data obtained by the fast sampling system [8]. These models do not contain TCA cycle and the fermentative pathways, and thus cannot simulate the typical aerobic batch culture.

In the present research, therefore, we considered several kinetic models for the TCA cycle, anapleorotic pathways as well as the glycolysis and the PP pathway to simulate the time profiles of the batch and continuous cultures. Moreover, most of the kinetic models developed so far can express only enzyme level regulation due to the change in the concentrations of substrate and product as well as various effectors. Thus, the application of the conventional model is limited in practice to some extent. Recently, several mathematical models which describe the effects of global regulators on the metabolic pathway reactions for catabolite repression for substrate uptake [9] and for *suc *mutant for glutamate production [10] have been proposed, which pay attention to particular pathways. Recently, we estimated the flux changes during batch culture of *E. coli *based on ^13^C-labeling experiment using CE-TOF/MS [11]. It is quite important to estimate the flux changes of the main metabolic pathways, allowing opportunity for the proper analysis of the energy metabolism and cell synthesis. Although ^13^C-metabolic flux analysis has proven to be quite useful [12,13], it is a method of the analysis of a static physiological state of the organism and does not have predictability characteristics. It is highly important and indeed useful to be able to predict cell growth characteristics. In the present study, therefore, we attempted to develop a new model for the cell growth rate with the advantages of considering the metabolic fluxes and an enzymatic model. Furthermore, incorporating the relationship between ATP production rate obtained by the intracellular fluxes of the main metabolic pathways and the cell growth rate together with some rule-based approach for gene-level regulation. Once we could simulate the whole main metabolic pathways, we may be able to compute CO_2 _production rate and NAD(P)H production rate as well as ATP production rate. In particular, we attempted to simulate several single-gene knockout mutants to show the utility of the model and its limitations. Some of the experiments were also conducted to verify the simulation result, which seems to be quite important for the practical use of modeling and simulation.

## Results

**Figure 1:**
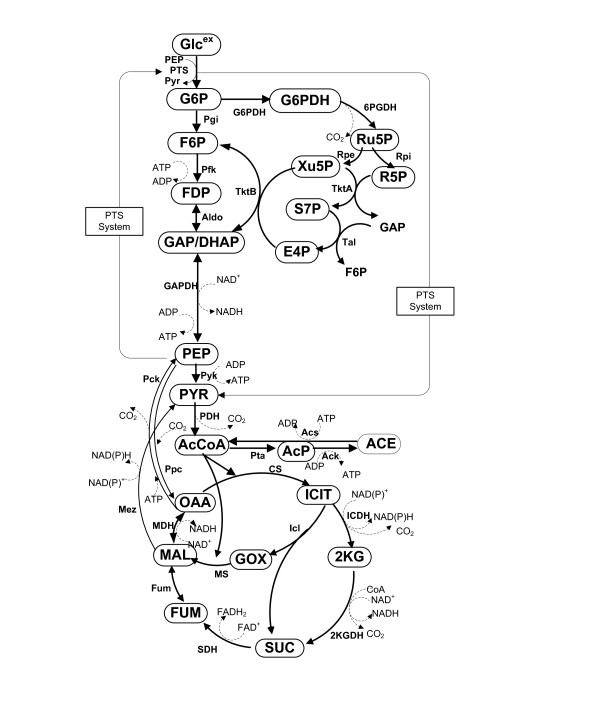
**Metabolic pathways considered in this simulation**.

In the present study, we considered the main metabolic pathways as given in Figure 1. Since most of the enzymatic reaction models have been developed based on in vitro kinetics as given in Table 1, some model parameters had to be retuned based on the intracellular metabolite concentrations measured in vivo. In the present simulation, we employed the model parameter values as given in the original literature except $v_{i}^{\max}$ values, where $v_{i}^{\max}$ was retuned based on the intracellular metabolite concentrations and the flux values obtained from the chemostat cultures [8,14]. The resulting model parameters used in the present simulation are listed in Table 2. The concentrations of cofactors such as CoA, ATP, ADP, AMP, NADH, NAD^+^, NADPH and NADP^+ ^were assumed to be constant [8], and the values used for the simulation are given in Table 3.

**Table 1:** Kinetic rate equations

Reactions	Model equations		References
Cell growth	$\mu =\{\begin{array}{c} \mu_{m}\left(1-\frac{\left[X\right]}{X_{m}}\right)\left(\frac{\left[Glc^{ex}\right]}{K_{s}+[Glc^{ex}]}\right)\;k_{ATP}\;v_{ATP}\left(.\right),\left([Glc^{ex}]>0\right)\\ \frac{\mu_{mA}[Ace^{ex}]}{K_{sA}+[Ace^{ex}]}k_{ATP}v_{ATP}(.),([Glc^{ex}]\leq \text{1}\;and\;[Ace^{ex}]>0) \end{array}$	(1a) (1b)	

Phosphotransferasesystem	$v_{PTS}=\frac{v_{PTS}^{\max}[Glc^{ex}]\frac{\left[PEP\right]}{\left[PYR\right]}}{\left(K_{a1}+K_{a2}\frac{\left[PEP\right]}{\left[PYR\right]}+K_{a3}[Glc^{ex}]+[Glc^{ex}]\frac{\left[PEP\right]}{\left[PYR\right]}\right)\left(1+\frac{{\left[G6P\right]}^{n_{G6P}}}{K_{G6P}}\right)}$	(2a)	[8,33]

Non-PTS glucokinase	$v_{Glk}=\frac{v_{Glk}^{\max}\;[Glc][ATP]}{\left(K_{m}^{Glc}+[Glc]\right)\left(K_{m}^{ATP}+[ATP]\right)}$	(2b)	[49]

Phosphoglucoseisomerase	$v_{Pgi}=\frac{v_{Pgi}^{\max}\left(\left[G6P\right]-\frac{\left[F6P\right]}{K_{eq}}\right)}{K_{G6P}\left(1+\frac{F6P}{K_{F6P}\left(1+\frac{\left[F6P\right]}{K_{6pginh}^{F6P}}\right)}+\frac{\left[6PG\right]}{K_{6pginh}^{G6P}}\right)+G6P}$	(3)	[8]

Phospho-fructokinase	$v_{Pfk}=\frac{v_{Pfk}^{\max}K_{ATP}\left[F6P\right]}{\begin{array}{c} K_{\left(ATP,ADP\right)}\left(\left[F6P\right]+K_{S}^{F6P}\frac{K_{b\left(ADP,AMP\right)}+\frac{\left[PEP\right]}{K_{PEP}}}{K_{a\left(ADP,AMP\right)}}\right)\times \\ \left(1+\frac{L_{Pfk}}{{\left(1+\left[F6P\right]\left(\frac{K_{a\left(ADP,AMP\right)}}{K_{s}^{F6P}\left(K_{b\left(ADP,AMP\right)}+\frac{\left[PEP\right]}{K_{PEP}}\right)}\right)\right)}^{n_{Pfk}}}\right) \end{array}}$	(4)	[8]

Adolase	$v_{Aldo}=\frac{v_{Aldo}^{\max}\left([FDP]-\frac{\left[GAP][GAP\right]}{K_{eq}}\right)}{\left(K_{FDP}+[FDP]+\frac{K_{GAP}[GAP]}{\left[K_{eq}V_{blf}\right]}+\frac{K_{DHAP}[GAP]}{\left[K_{eq}V_{blf}\right]}+\frac{\left[FDP][GAP\right]}{K_{in\;h}^{PEP}}+\frac{\left[GAP][GAP\right]}{K_{eq}V_{blf}}\right)}$	(5)	[8]

*G3P dehydrogenase*	$v_{GAPDH}=\frac{v_{GAPDH}^{\max}\left([GAP]-\frac{\left[PEP][NADH\right]}{K_{eq}[NAD]}\right)}{\left(K_{GAP}\left(1+\frac{\left[PEP\right]}{K_{PGP}}\right)+[GAP]\right)\left(\frac{K_{NAD}}{NAD}\left(1+\frac{\left[NADH]\right|}{K_{NADH}}\right)+1\right)}$	(6)	[8]

*Pyruvate kinase*	$v_{Pyk}=\frac{v_{Pyk}^{\max}[PEP]{\left(\frac{\left[PEP\right]}{K_{PEP}}+1\right)}^{n_{Pyk}-1}[ADP]}{K_{PEP}\left(L_{Pyk}{\left(\frac{1+\frac{\left[ATP\right]}{K_{ATP}}}{\frac{\left[FDP\right]}{K_{FDP}}+\frac{\left[AMP\right]}{K_{AMP}}+1}\right)}^{n_{Pyk}}+{\left(\frac{\left[PEP\right]}{K_{PEP}}+1\right)}^{n_{Pyk}}\right)\left([ADP]+K_{ADP}\right)}$	(7)	[8]

PEP carboxylase	$v_{Ppc}=\frac{K_{1}+K_{2}[AcCoA]+K_{3}[FDP]+K_{4}[AcCoA][FDP]}{1+K_{5}[AcCoA]+K_{6}[FDP]}\left(\frac{\left[PEP\right]}{K_{m}+[PEP]}\right)$	(8)	[38]

PEP carboxykinase	$v_{Pck}=v_{Pck}^{\max}\left(\frac{[OAA]\frac{\left[ATP\right]}{\left[ADP\right]}}{\begin{array}{c} K_{m}^{OAA}\frac{\left[ATP\right]}{\left[ADP\right]}+[OAA]\frac{\left|[ATP\right]}{\left[ADP\right]}+\frac{K_{i}^{ATP}K_{m}^{OAA}}{K_{i}^{ADP}}+\frac{K_{i}^{ATP}K_{m}^{OAA}}{K_{m}^{PEP}K_{i}^{{}^{ADP}}}[PEP]+\\ \frac{K_{i}^{ATP}K_{m}^{OAA}}{K_{i}^{PEP}K_{I}^{ATP}}\frac{\left[ATP][PEP\right]}{\left[ADP\right]}+\frac{K_{i}^{ATP}K_{m}^{OAA}}{K_{i}^{ADP}K_{I}^{OAA}}[OAA] \end{array}}\right)$	(9)	[15]

Pyruvate dehydrogenasecomplex	$v_{PDH}=\frac{\frac{v_{PDH}^{\text{max}}}{\left[NAD\right]}\left(\frac{1}{1+K_{i}\frac{\left[NADH\right]}{\left[NAD\right]}}\right)\left(\frac{\left[PYR\right]}{K_{m}^{PYR}}\right)\left(\frac{1}{K_{m}^{NAD}}\right)\left(\frac{\left[COA\right]}{K_{m}^{COA}}\right)}{\left(1+\frac{\left[PYR\right]}{K_{m}^{PYR}}\right)\left(\frac{1}{NAD}+\frac{1}{K_{m}^{NAD}}+\frac{\left[NADH\right]}{K_{m}^{NADH}[NAD]}\right)\left(1+\frac{\left[COA\right]}{K_{m}^{COA}}+\frac{\left[AcCoA\right]}{K_{m}^{AcCoA}}\right)}$	(10)	[40]

Pta	$v_{Pta}=\frac{v_{Pta}^{\text{max}}\left(\frac{1}{K_{i}^{AcCoA}K_{m}^{P}}\right)\left([AcCoA][P]-\frac{\left[AcP][CoA\right]}{K_{eq}}\right)}{\left(1+\frac{\left[AcCoA\right]}{K_{i}^{AcCoA}}+\frac{\left[P\right]}{K_{i}^{P}}+\frac{AC\text{P}}{K_{i}^{ACP}}+\frac{\left[CoA\right]}{K_{i}^{CoA}}+\left(\frac{\left[AcCoA][P\right]}{K_{i}^{AcCoA}K_{m}^{P}}\right)+\left(\frac{\left[AcP][CoA\right]}{K_{m}^{ACP}K_{i}^{CoA}}\right)\right)}$	(11)	[40,41]

Ack	$v_{Ack}=\frac{v_{Ack}^{\max}\left(\frac{1}{K_{m}^{ADP}K_{m}^{ACP}}\right)\left(\left[AcP\right]\left[ADP\right]-\frac{\left[ACE\right]\left[ATP\right]}{K_{eq}}\right)}{\left(1+\frac{\left[AcP\right]}{K_{m}^{ACP}}+\frac{ACE}{K_{m}^{ACE}}\right)\left(1+\frac{\left[ADP\right]}{K_{m}^{ADP}}+\frac{\left[ATP\right]}{K_{m}^{ATP}}\right)}$	(12)	[40]

Acs	$v_{Acs}=\frac{v_{Acs}^{\text{max}}[ACE][NADP]}{\left(K_{m}+[ACE])(K_{eq}+[NADP]\right)}$	(13)	[42]

Citrate Synthase	$v_{CS}=\frac{v_{CS}^{\max}[AcCoA][OAA]}{\begin{array}{c} \left(K_{d}^{AcCoA}K_{m}^{OAA}+K_{m}^{AcCoA}[OAA]\right)+\left([AcCoA]K_{m}^{OAA}\left(1+\frac{\left[NADH\right]}{K_{i1}^{NADH}}\right)\right)+\\ \left([AcCoA][OAA]\left(1+\frac{\left[NADH\right]}{K_{i2}^{NADH}}\right)\right) \end{array}}$	(14)	[43]

*ICDH*	$v_{ICDH}=\frac{\left[ICDH\right]\frac{K_{f}}{K_{m}^{iCiT}}K_{d}^{NADP}\left(\left[ICIT\right]-\frac{\left[NADPH\right]\left[2KG\right]}{K_{eq}^{ICDH}\left[NADP\right]}\right)}{\left(\begin{array}{l} \frac{1}{\left[NADP\right]}+\frac{\left[ICIT\right]K_{m}^{NADP}}{K_{m}^{iCiT}K_{d}^{NADP}\left[NADP\right]}+\frac{1}{K_{d}^{NADP}}+\frac{\left[ICIT\right]}{K_{m}^{iCiT}K_{d}^{NADP}}+\\ \frac{\left[ICIT\right]}{K_{d}^{iCiT}\left[NADP\right]}\frac{\left[NADPH\right]K_{m}^{NADP}}{K_{m}^{iCiT}K_{d}^{NADP}K_{ein\;h}^{NADPH}}+\frac{\left[NADPH\right]K_{ekn\;h}^{2\;KG}}{K_{m}^{2\;KG}K_{en\;he}^{NADPH}\left[NADP\right]}+\frac{\left[2KG\right]K_{m}^{NADPH}}{K_{m}^{2KG}K_{en\;he}^{NADPH}\left[NADP\right]}+\\ \frac{\left[2KG\right]}{K_{m}^{2KG}}\frac{\left[NADPH\right]}{K_{en\;he}^{NADPH}\left[NADP\right]}+\frac{\left[2KG\right]K_{m}^{NADPH}}{K_{m}^{2KG}K_{en\;he}^{NADPH}}\frac{\left[NADPH\right]}{K_{ekn}^{NADP}\left[NADP\right]} \end{array}\right)}$	(15)	[44]

Isocitrate lyase	$v_{Icl}=\frac{v_{Icl\_f}^{\text{max}}\frac{\left[ICIT\right]}{K_{m}^{iCiT}}}{\left(1+\frac{\left[ICIT\right]}{K_{m}^{iCiT}}+\frac{\left[SUC\right]}{K_{m}^{SUC}}+\frac{\left[GOX\right]}{K_{m}^{GOX}}+\frac{\left[ICIT\right]}{K_{m}^{iCiT}}\frac{\left[SUC\right]}{K_{m}^{SUC}}+\frac{\left[SUC\right]}{K_{m}^{SUC}}\frac{\left[GOX\right]}{K_{m}^{GOX}}+\frac{I}{K_{I}}\right)}$	(16)	[46]

Malate synthase	$v_{MS}=\frac{v_{MS\_f}^{\text{max}}\frac{\left[GOX\right]}{K_{m}^{GOX}}\frac{\left[AcCoA\right]}{K_{m}^{AcCoA}}-v_{MS\_r}^{\max}\frac{\left[MAL\right]}{K_{m}^{MAL}}}{\left(1+\frac{\left[GOX\right]}{K_{m}^{GOX}}+\frac{\left[MAL\right]}{K_{m}^{MAL}}+\left(1+\frac{\left[AcCoA\right]}{K_{m}^{AcCoA}}\right)\right)}$	(17)	[46]

αKG dehydrogenase	$v_{\alpha K\text{G}DH}=\frac{v_{2KGDH}^{\max}[\alpha KG][CoA]}{\left\{\begin{array}{c} \frac{K_{m}^{NAD}[\alpha KG][CoA]}{\left[NAD\right]}+K_{m}^{CoA}[\alpha KG]+K_{m}^{2K\text{G}}[CoA]+[\alpha KG][CoA]+\\ \frac{K_{m}^{2K\text{G}}K_{z}[SUC][NADH]}{K_{I}^{{}^{SUC}}[NAD]}+\frac{K_{m}^{NAD}[\alpha KG][CoA][NADH]}{K_{I}^{NADH}[NAD]}+\\ \frac{K_{m}^{CoA}[\alpha KG][SUC]}{K_{I}^{SUC}}+\frac{K_{m}^{2K\text{G}}K_{Z}[\alpha KG][SUC][NADH]}{K_{I}^{{}^{2K\text{G}}}K_{I}^{{}^{SU\text{C}}}[NAD]} \end{array}\right\}}$	(18)	[45]

Succinate dehydrogenase	$v_{SDH}=\frac{v_{SDH1}v_{SDH2}\left([SUC]-\frac{\left[FUM\right]}{K_{eq}}\right)}{K_{m}^{SUC}v_{SDH2}+v_{SDH2}[SUC]+\frac{V_{SDH1}[FUM]}{K_{eq}}}$	(19)	[45]

Fumarase	$v_{Fum}=\frac{v_{Fum1}v_{Fum2}\left(\left[FUM\right]-\frac{\left[MAL\right]}{K_{Fumeq}}\right)}{K_{m}^{Fum}v_{Fum1}+v_{Fum2}\left[FUM\right]+\frac{V_{Fum1}\left[MAL\right]}{K_{eq}}}$	(20)	[45]

Malate dehydrogenase	$v_{MDH}=\frac{v_{MDH}{}_{1}v_{MDH}{}_{2}\left(\left[MAL\right]-\frac{\left[OAA\right]}{K_{eq}}\right)}{\left(\begin{array}{l} \frac{K_{I}^{NAD}K_{m}^{MAL}v_{MDH2}}{\left[NAD\right]}+K_{m}^{MAL}v_{MDH2}+\frac{K_{m}^{NAD}V_{MDH2}\left[MAL\right]}{\left[NAD\right]}+v_{MDH2}\left[MAL\right]+\\ \frac{K_{m}^{OAA}v_{MDH1}\left[NADH\right]}{K_{eq}\left[NAD\right]}+\frac{K_{m}^{NADH}v_{MDH1}\left[OAA\right]}{K_{eq}\left[NAD\right]}+\frac{v_{MDH1}\left[NADH\right]\left[OAA\right]}{K_{eq}\left[NAD\right]}+\\ \frac{v_{MDH1}K_{m}^{OAA}\left[NADH\right]}{K_{eq}K_{I}^{NAD}}+\frac{v_{MDH2}K_{m}^{NAD}\left[MAL\right]\left[OAA\right]}{K_{I}^{OAA}\left[NAD\right]}+\frac{v_{MDH2}\left[MAL\right]\left[NADH\right]}{K_{I}^{NADH}}+\\ \frac{v_{MDH1}\left[MAL\right]\left[NADH\right]\left[OAA\right]}{K_{eq}K_{I}^{MAL}\left[NAD\right]}+\frac{v_{MDH2}\left[MAL\right]\left[OAA\right]}{K_{II}^{OAA}}+\frac{v_{MDH1}\left[NADH\right]\left[OAA\right]}{K_{II}^{NAD}K_{eq}}+\\ \frac{K_{I}^{NAD}v_{MDH2}\left[MAL\right]\left[NADH\right]\left[OAA\right]}{K_{II}^{NAD}K_{m}^{OAA}K_{I}^{NADH}} \end{array}\right)}$	(21)	[45]

Malic enzyme	$v_{Mez}=\frac{v_{Mez}^{\max}[MAL][NADP]}{\left(K_{MAL}+[MAL])(K_{eq}+[NADP]\right)}$	(22)	[45]

G6PDH	$v_{G6PDH}=\frac{v_{G6PDH}^{\max}\left[G6P\right]}{([G6P]+K_{G6P})\left(1+\frac{\left[NADPH\right]}{K_{NADPH}^{G6P}}\right)\left(\frac{K_{NADP}}{\left[NADP\right]}\left(1+\frac{NADPH}{K_{NADPH}^{NADP}}\right)+1\right)}$	(23)	[8]

PGDH	$v_{G6PDH}=\frac{v_{G6PDH}^{\max}\left[G6P\right]}{([6PG]+K_{6PG}\text{)}\left(1+\frac{K_{NADP}}{NADP}\left(1+\frac{\left[NADPH\right]}{K_{NADPH}^{inh}}\right)\left(\text{1+}\frac{\left[ATP\right]}{K_{ATP}^{inh}}\right)\right)}$	(24)	[8]

Rpe	$v_{Rpe}=v_{Rpe}^{\text{max}}\left([Ru5P]-\frac{\left[Xu5P\right]}{K_{eq}^{Rpe}}\right)$	(25)	[8]

Rpi	$v_{Rpi}=v_{Rpi}^{\max}\left([Ru5P]-\frac{\left[R5P\right]}{K_{eq}^{Rpi}}\right)$	(26)	[8]

TktA	$v_{TktA}=v_{TktA}^{\text{max}}\left([R5P][Xu5P]-\frac{\left[S7P][GAP\right]}{K_{eq}^{TktA}}\right)$	(27)	[8]

TktB	$v_{TktB}=v_{TktB}^{\text{max}}\left([Xu5P][E4P]-\frac{\left[F6P][GAP\right]}{K_{eq}^{TktB}}\right)$	(28)	[8]

Tal	$v_{Tal}=v_{Tal}^{\text{max}}\left([GAP][S7P]-\frac{\left[E4P][F6P\right]}{K_{eq}^{TktB}}\right)$	(29)	[8]

**Table 2:** Model parameters

Enzyme	Kinetic parameter values	Original source
Cell growth	*μ_m _= *0.6, *K*_*s *_= 0.1, *μ*_*mA *_= 0.9, *K_sA _= 0.01*, *X_m _= *2.3	estimated

Glk	$v_{Glk}^{\max}=4.503mmol/gDCW.h,K_{m}^{GLC}=0.12mM,K_{m}^{AATP}=0.5mM$	[49]

PTS	$v_{PTS}^{\max}=25.739mmol/gDCW.h,K_{a1}=1.0mM,K_{a2}=0.01\text{m}M\text{, }K_{a3}=1.0,\;n_{G6P}=4$, *K*_*G*6*p *_= 0.5*mM*	[8,33]

Pgi	$v_{PGI}^{\text{max}}=26.3711mmol/gDCW.h,K_{\text{G}6P}=2.46mM,\;K_{F6P}=0.2mM,K_{eq}=0.43mM$, $K_{6pginh}^{{}^{G6P}}=0.2mM,K_{6pginh}^{{}^{F6P}}=0.2mM$	[8]

Pfk	$v_{Pfk}^{\max}=24.613mmol/gDCW.h,K_{\left(ATP\right)}=4.27mM,K_{\left(ATP\text{,}ADP\right)}=4.6944\text{m}M$, $K_{a(ADP,AMP)}=1.1118\text{m}M,\;K_{b(ADP,AMP)}=98.88mM,n_{Pfk}=4,L_{Pfk}=1000,K_{s}^{F6P}=0.14mM$, *K*_*PEP *_= 3.26*mM*	[8]

Aldo	$v_{Aldo}^{\text{max}}=2.8337mmol/gDCW.h,K_{FDP}=0.133\text{m}M,K_{\text{G}AP}=0.088\text{m}M,K_{DHAP}=0.088\text{m}M$, $K_{inh}^{\text{G}AP}=0.6mM,K_{eq}=0.14mM,V_{blf}=2$	[8]

GAPDH	$v_{GAPDH}^{\text{max}}=121.29mmol/gDCW.h,K_{\text{G}AP}=0.15mM,K_{PGP}=0.1mM,K_{NAD}=0.45mM$, *K*_*NADH *_= 0.02m*M*, *K*_*eq *_= 0.63	[8]

Pyk	$v_{Pyk}^{\text{max}}=1.0849mmol/gDCW.h,K_{PEP}=0.31mM,K_{FDP}=0.19mM,K_{AMP}=0.2mM,n_{Pyk}=4$ *K*_*ATP *_= 22.5*mM*, *K*_*ADP *_= 0.26*mM*, *L*_*Pyk *_= 1000,	[8]

PDH	$v_{PDH}^{\max}=27171mmol/gDCW.h,K_{m}^{PYR}=1mM,K_{m}^{NAD}=0.4mM,K_{m}^{AcCoA}=0.008mM$, $K_{m}^{NADH}=0.1mM,K_{m}^{\text{C}OA}=0.014mM,K_{i}^{PDH}=46.4mM$	[40]

CS	$v_{CS}^{\max}=17.36mmol/gDCW.h,K_{d1}^{H}=0.00001mM,K_{d2}^{H}=0.0002mM,K_{m}^{AcCoA}=0.18mM$, $K_{m}^{OAA}=0.04mM,K_{d}^{AcCoA}=0.1mM,K_{i}^{ATP}=0.58mM,K_{\iota 1}^{2KG}=0.015mM,K_{i2}^{2KG}=0.256\text{m}M$, $K_{i1}^{NADH}=0.00033mM,K_{i2}^{NADH}=0.0084mM,K_{cat0}=1$	[43]

ICDH	$v_{ICDH}^{\max}=24.42mmol/gDCW.h,K_{eq}=1000mM,k_{f}=4830-1/\text{min},K_{m}^{2kG}=0.038mM$, $K_{m}^{iCiT}=0.0059mM,K_{d}^{NADP}=0.0013mM,K_{m}^{NADP}=0.0227mM,K_{d}^{NADH}=0.12mM$, $K_{d}^{iCiT}=0.03mM,K_{einh}^{NADPH}\;=0.007mM,K_{eknh}^{\text{2K}G}=5.5mM,K_{d}^{co_{2}}=1.6mM,K_{eke}^{co_{2}}=1.6mM$, $K_{ekn}^{NADP}=0.00016mM,K_{m}^{NADPH}=0.0036mM,K_{enhe}^{NADPH}=0.028mM$	[44]

Icl	$V_{Icl_{-}f}^{\text{max}}=3.8315mmol/gDCW.h,v_{Icl\_r}^{\max}=2.585/100mmol/gDCW.h,K_{m}^{iCiT}=0.604mM$, $K_{m}^{SUC}=0.59mM,K_{m}^{GOX}=0.13mM,K_{I}^{{}^{ICL}}=0.003mM$	[46]

MS	$K_{MS\_f}^{\max}=3.6968mmol/gDCW.h,K_{MS\_r}^{\text{max}}=13.742/100mmol/gDCW.h,K_{m}^{\text{G}OX}=2mM$, $K_{m}^{AcCoA}=0.01mM,K_{m}^{MAL}=1mM,K_{m}^{CoA}=0.1mM$	[46]

2KGDH	$v_{2KGDH}^{\max}=137.435mmol/gDCW.h,K_{m}^{\text{2}KG}=1.0mM,K_{I}^{2KG}=0.75mM,K_{m}^{CoA}=0.002mM$, $K_{m}^{NAD}=0.07mM,K_{m}^{SUC}=1.0mM,K_{m}^{NADH}=0.018mM,K_{Z}=1.5$	[45]

SDH	$K_{m}^{SUC}=0.1,v_{SDH1}=1.1334mmol/gDCW.h,v_{SDH2}=1.1334mmol/gDCW.h,K_{eq}=10$	[45]

MDH	$v_{MDH1}=25.874mmol/gDCW.h,v_{MDH2}=25.874mmol/gDCW.h,K_{eq}=1.0,K_{I}^{NAD}=0.31mM$, $K_{I}^{NADH}=0.04mM,K_{I}^{MAL}=3.30mM,K_{I}^{OAA}=0.27mM,K_{m}^{NAD}=0.1mM,K_{m}^{NADH}=0.04mM$, $K_{m}^{MA\text{L}}=1.33mM,K_{m}^{OAA}=0.27mM,K_{II}^{NAD}=0.31mM,K_{II}^{OAA}=0.17mM$	[45]

FUM	$v_{FUM1}=1.1334mmol/gDCW.h,V_{FUM2}=1.1334mmol/gDCW.h,K_{eq}=10,K_{m}^{FUM}=0.1mM$	[45]

Ppc	$v_{Ppc}^{\max}=0.1885mmol/gDCW.h,K_{m}^{PEP}=0.323lmM,k_{1}=0.03176,k_{2}=1.2878mM$, *k*_3 _= 0.0542*mM*, *k*_4 _= 0.8139*mM*, *k*_5 _= 0.0939*mM*, *k*_6 _= 0.2693*mM*	[38]

Pck	$v_{Pck}^{\max}=4.5116mmol/gDCW.h,K_{m}^{ATP}=0.06\text{m}M,K_{I}^{ATP}=0.04mM,K_{i}^{ATP}=0.04mM$, $K_{m}^{OAA}=0.67mM,K_{i}^{PEP}=0.06mM,K_{I}^{OAA}=0.67mM,K_{m}^{PEP}=0.07mM,K_{i}^{ADP}=0.04mM$	[15]

Mez	$v_{Mez}^{\max}=0.069945mmol/gDCW.h,K_{m}^{MAL}=0.37mM,K_{eq}=0.10$	[45]

Pta	$v_{Pta}^{\text{max}}=12.585mmol/gDCW.h,K_{i}^{AcCoA}=0.2mM,K_{m}^{P}=2.6mM,K_{i}^{P}=2.6mM$, $K_{m}^{ACP}=0.7mM,K_{i}^{ACP}=0.2mM,K_{i}^{coA}=0.029mM,K_{eq}=0.0281mM$	[40]

Ack	$v_{Ack}^{\max}=2865.3mmol/gDCW.h,K_{m}^{ACP}=0.16mM,K_{m}^{ADP}=0.5mM,K_{m}^{ACE}=7mM$, $K_{m}^{ATP}=0.07mM,K_{eq}=174.217mM$	[40]

Acs	$v_{Acs}^{\text{max}}=17.9068mmol/gDCW.h\;K=0.089971mmol/gDCW.h,\;K_{m}=0.07mM$	[42]

G6PDH	$v_{\text{G}6PDH}^{\text{max}}=0.97922mmol/gDCW.h,K_{G6P}=14.4mM\text{, }K_{NADP}=0.015mM,K_{NADPH}^{NADP}=0.01mM,$ $K_{NADPH}^{G6P}=0.18mM$	[8]

6PGDH	$v_{6GPDH}^{\text{max}}=1.81mmol/gDCW.h,K_{G6P}=0.1mM,K_{NADP}=0.028mM,K_{NAD}{}_{Pinh}=0.01mM$, *K*_*ATPinh *_= 3.0*mM*	[8]

Rpe	$v_{Rpe}^{\max}=18.485mmol/gDCW.h,K_{eq}^{Rpe}=1.4mM$	[8]

Rpi	$v_{Rpi}^{\max}=13.318mmol/\;gDCW.h\;K_{eq}^{Rpi}=4.0mM$	[8]

TktA	$v_{TktA}^{\max}=29.348mmol/gDCW.h,K_{eq}^{TktA}=1.2mM$	[8]

TktB	$v_{TKtB}^{\max}=316.22mmol/gDCW.h,K_{eq}^{TktB}=10.0mM$	[8]

Tal	$v_{Tal}^{\max}=24.499mmol/gDCW\text{.}h,K_{eq}^{Tal}=1.05mM$	[8]

**Table 3:** Cofactor concentrations

Cofactor	Concentrations	Original source
ADP	0.595 mM	[8]
AMP	0.955 mM	[8]
ATP	4.27 mM	[8]
CoA	0.001mM	
NAD	1.47 mM	[8]
NADH	0.1 mM	[8]
NADP	0.195 mM	[8]
NADPH	0.062 mM	[8]
P	10 mM	[40]

**Figure 2:**
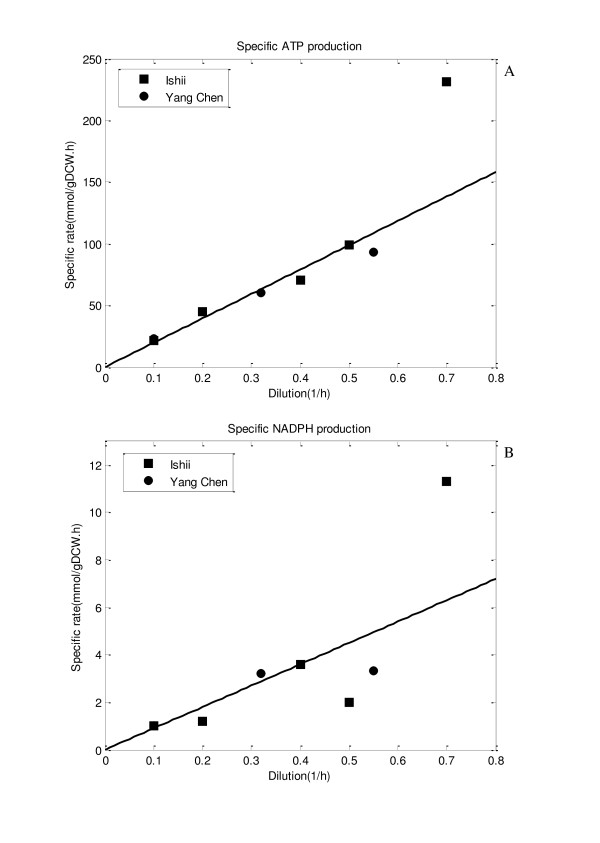
**The specific ATP and NADPH production rates with respect to dilution rate in the continuous culture**: (A) the specific ATP production rate vs. dilution rate; (B) the specific NADPH production rate vs. dilution rate. The rectangle symbols are the experimental data from [14] while circle symbol are the experimental data from [15].

**Figure 3:**
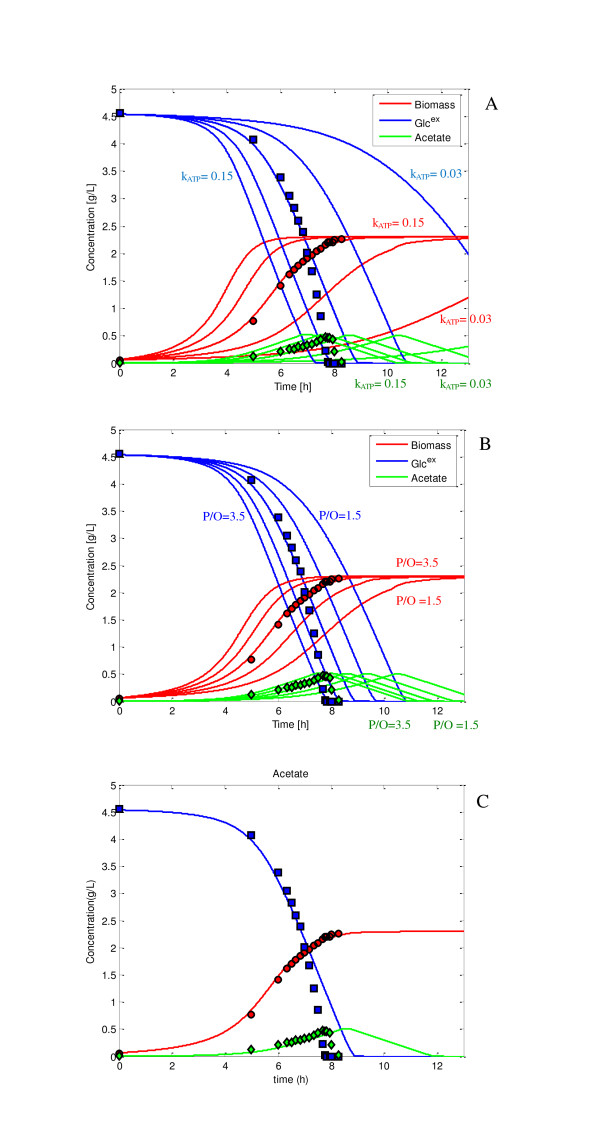
**Effect of *k*_*ATP *_and *(P/O) *ratio on the cell growth characteristics**: (A) Effect of *k*_*ATP *_; (B) effect of (P/O) ratio; (C) cell growth characteristics for the best fitted value where *k*_*ATP *_= 0.10 and (*P/O*) = 2.5.

Figure 2a shows the relationship between the specific growth rate and the specific ATP production rate computed by Eq. (3) as shown in Materials and Methods section based on the flux data previously obtained for the wild type *E. coli *based on ^13^C-labeling experiments [14,15]. Figure 2a indicates that the specific ATP production rate is linearly correlated with the specific growth rate. Figure 3 shows the batch cultivation result for the wild type *E. coli*, where the lines are the simulation result, while the symbols are the experimental data. Figure 3a shows the effect of *k*_*ATP *_, while Figure 3b shows the effect of (*P*/*O*) ratio on the cultivation characteristics, where *k*_*ATP *_and (*P*/*O*) ratio are the model parameters as shown in Eqs. (4a), (4b) and (5) in Materials and Methods section. In past simulations or the analysis of ATP production, (*P*/*O*) ratio had been assumed to be a certain value. Since the assumed value may be different in practice, here we considered it to be a model parameter to be fitted to the experimental data. Figure 3c shows the simulation result where *k*_*ATP *_and (*P*/*O*) ratio were fitted to the experimental data, yielding values: *k*_*ATP *_= 0.09 and (*P*/*O*) ratio = 2.5.

During the fitting procedure, it was found that some of the initial concentrations of the intracellular metabolites such as OAA, PEP and PYR affected the growth cultivation characteristics. Additional file 1a shows the effect of initial OAA concentration, while Additional file 1b shows the effect of initial PEP concentration on the fermentation characteristics. Since those will change depending on the pre-cultivation, we assumed those to be also adjustable parameters for the simulation of batch culture. Note that initial OAA concentration was low around 0.01 mmol/gDCW, while initial PEP concentration was around 0.1 mmol/gDCW. The low value of intracellular OAA concentration coincides with the fact that anapleorotic pathway is required for OAA for cell synthesis.

The changes in the intracellular metabolite concentrations for batch culture of wild type as shown in Figure 3c are given in Additional file 2, indicating that as glucose is consumed, the concentrations of metabolites G6P/F6P, FDP and GAP/DHAP increase. After glucose depletes these metabolite concentrations decreased sharply, however for PEP concentration, PTS flux became zero, and thus PEP concentration tended to increase for short a period. Unlike other glycolysis intermediates as mentioned above, the PEP concentration slowly decreased. This is due to the exchange with OAA via Ppc and Pck. The change in PYR concentration affected AcCoA concentration, where the latter increased after glucose was depleted. The concentrations of the TCA cycle intermediates changed in a similar fashion. The glyoxylate pathway was activated after the glucose concentration became low level due to rule 6 of Additional file 3: Table S1.

**Figure 4:**
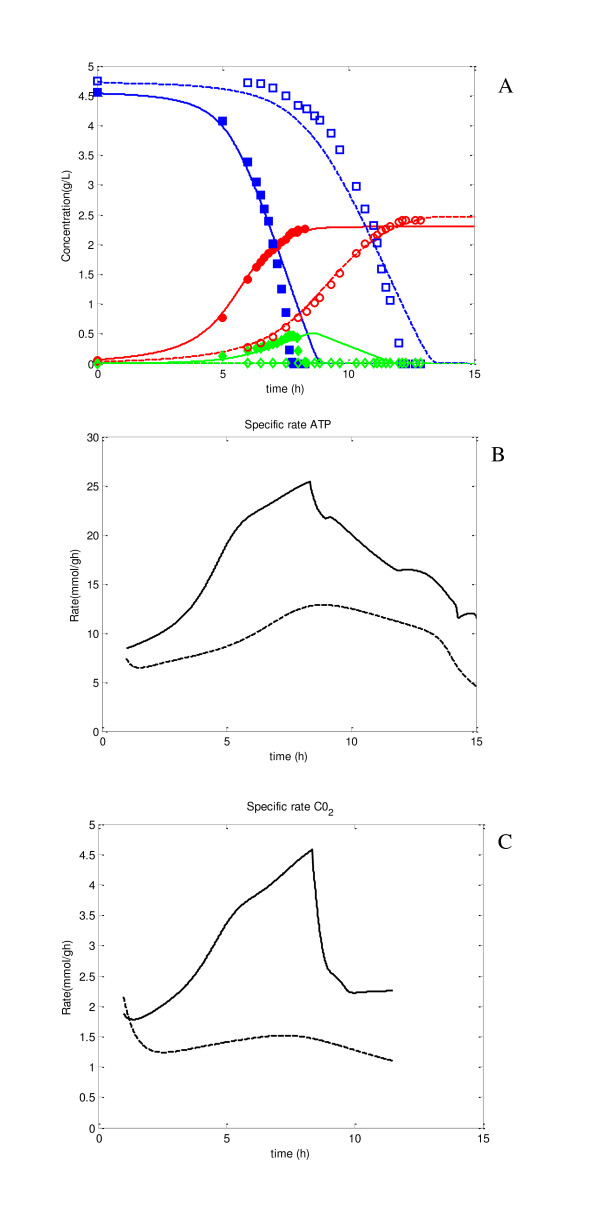
**Effect of Ppc knockout on fermentation characteristics, where solid lines represent the simulation result for the wild type, and dotted lines represent the simulation result for of Ppc knockout mutant**. The filled symbols represent experimental data for wild type, open symbols are for Ppc knockout mutant: (A) fermentation characteristics; (B) specific ATP production rate; (C) specific CO_2 _production rate.

From the point of view of practical applications, the primary interest is the predictability of the model for specific gene knockouts. Figure 4a shows the simulation result for the case of Ppc knockout (broken lines) as compared to the wild type (solid lines), where the filled symbols are the experimental data for the wild type, and the open symbols are those for Ppc mutant. Figure 4a shows reasonably good predictability of the simulation result. This is due to the incorporation of ATP-dependent specific growth rate as explained in Materials and Methods section. It could not be succeeded without its consideration. As illustrated in Additional file 2, upon Ppc knockout, PEP concentration increased, which in turn caused *v*_Pyk _to be increased, and thus PYR concentration increased. This lead to the accumulation of AcCoA, increasing its concentration, which was further, contributed towards by the fact that v_CS _flux had become lower. This is due to lower OAA concentration caused by Ppc knockout indicating that Ppc mutant has difficulty in growing in synthetic medium, whereas it can grow if LB medium was used for the pre culture since those contain amino acids etc., which can back up OAA [16]. This simulation result indicates the importance of the anapleorotic route of Ppc to aid in the backup of OAA. The significant decrease in OAA concentration caused significant decrease in TCA cycle fluxes or the activation of glyoxylate pathway, and these caused lower ATP production (Figure 4b), which then caused lower cell growth rate. It should also be noted that the overall cell yield or the final cell concentration for Ppc mutant was higher as compared to the wild type, consistent with experimental data (Figure 4a). The change in the specific CO_2 _production rate $\left(q_{co_{2}}\right)$ was also shown in Figure 4c. Note that the specific CO_2 _production rate may be measured by CO_2_/O_2 _off-gas analyzer, but it is usually less accurate. Moreover, the specific ATP production rate cannot be measured. Those can be estimated as shown in Figure 4b, c, and by comparing the values of wild type and a specific gene knockout mutant, may indeed give useful information.

**Figure 5:**
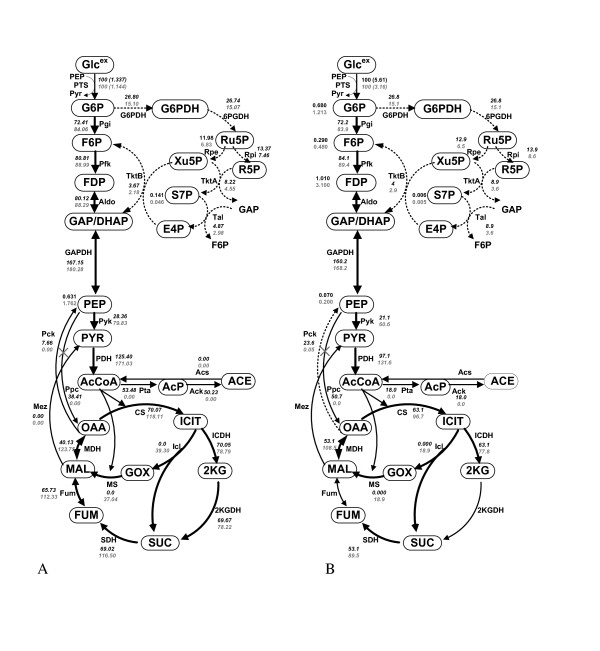
**Effect of Ppc knockout on the fluxes at the dilution rate of 0.2 h^-1 ^in the continuous culture, where bold lines indicates up-regulation and dotted line show down-regulation**. The upper figure for the wild type and the lower figure for the Ppc knockout mutant: (A) simulation result of Ppc knockout mutant as compared to wild type; (B) experimental result from [16].

The steady state flux values were also compared between the wild type and Ppc mutant at the dilution rate of 0.2 h^-1^, and the result is shown in Figure 5a, where the transient changes were also given in Additional file 4. Note that the flux through G6PDH was computed based on the specific NADPH production rate, *v*_*NADPH *_, found using Eqs.(7) and (8), where *k *_*NADPH *_was 13.5 from Figure 2b. Overall, a similar feature as state above for the batch culture can also be seen in continuous culture. Although the magnitudes may be somewhat different, the essential characteristics are consistent with the flux values obtained by ^13^C-labeling experiment as given by Figure 5b.

**Figure 6:**
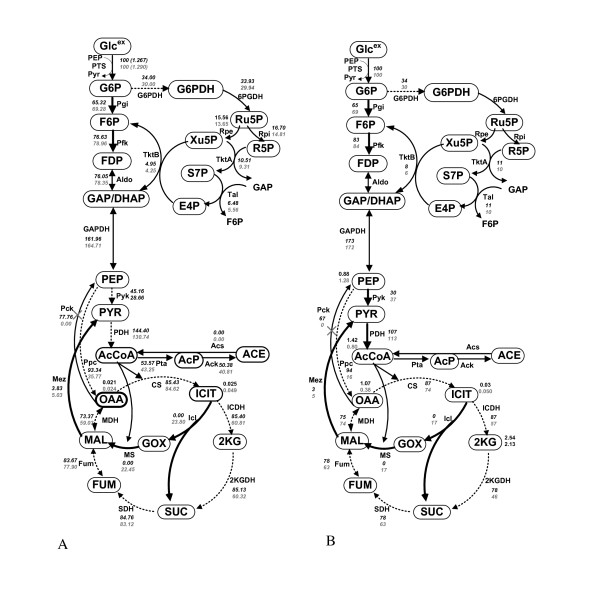
**Effect of Pck knockout on the fluxes at the dilution rate of 0.2 h^-1 ^in the continuous culture, where bold lines indicates up-regulation and dotted line show down-regulation**. The upper figure for the wild type and the lower figure for the Pck knockout mutant: (A) simulation result of Pck knockout mutant as compared to wild type; (B) experimental result from [15].

Figure 6 shows the comparison of the steady state fluxes between wild type and its Pck mutant, where Figure 6a shows the simulation result and Figure 6b shows the flux data computed based on ^13^C labelling experiment. The transient simulation result is given in Additional file 5, where the rule 6 of Additional file 3: Table S1 was activated in the simulation [15]. The simulation result indicates that the cell growth characteristics may be less affected as compared to the case of Ppc mutant, shown by experiment [15].

**Figure 7:**
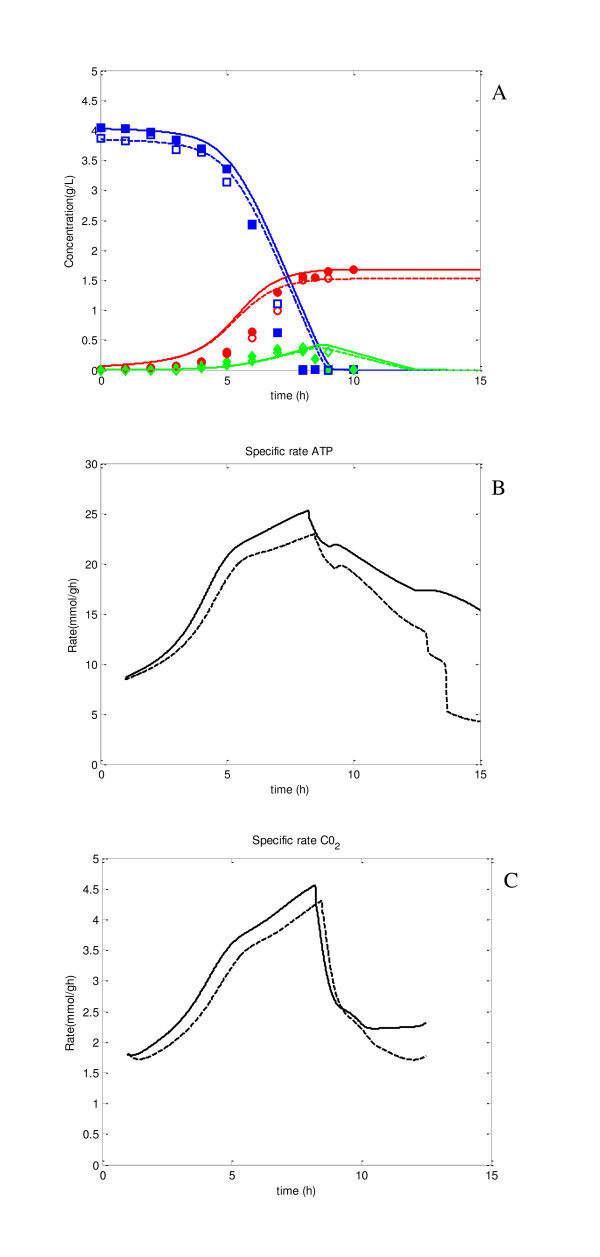
**Effect of Pyk knockout on fermentation characteristics, where solid lines represent the simulation result for the wild type, and dotted lines represent the simulation result for of Pyk knockout mutant**. The filled symbols represent experimental data for wild type, open symbols are for Pyk knockout mutant: (A) fermentation characteristics; (B) specific ATP production rate; (C) specific CO_2 _production rate.

**Figure 8:**
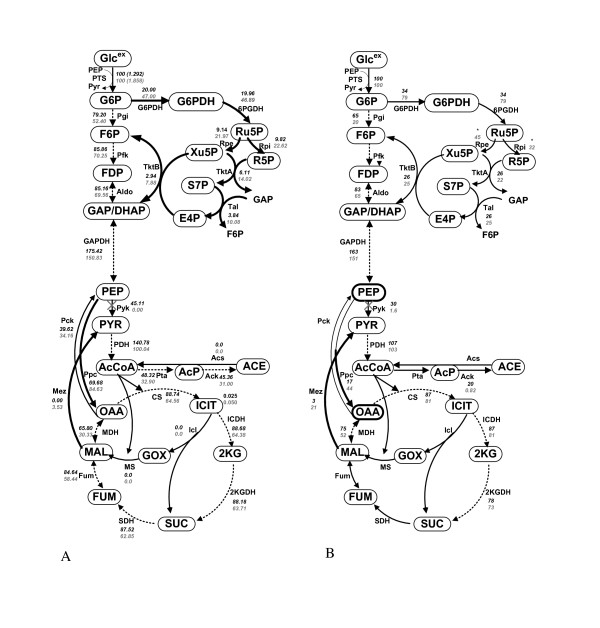
**Effect of Pyk knockout on the fluxes at the dilution rate of 0.2 h^-1 ^in the continuous culture, where bold lines indicates up-regulation and dotted line show down-regulation**. The upper figure for the wild type and the lower figure for the Pyk knockout mutant: (A) simulation result of Pyk knockout mutant as compared to wild type; (B) experimental result from [17].

Another simulation was conducted for Pyk knockout mutant as compared to the case of the wild type. As shown in Figure 7, the batch cultivation profile of the mutant changed little as compared to the wild type. However, the intracellular metabolite concentrations and the fluxes inside the cell are quite different from the case of the wild type as shown in Additional file 6. Figure 8a shows the comparison of the fluxes between the wild type and the Pyk mutant for the continuous culture at the dilution rate of 0.2 h^-1^, where the transient simulation result is given in Additional file 7. The up and down regulations of the fluxes are similar to the experimental data obtained by ^13^C-labeling experiments as shown in Figure 8b[17]. The result indicates that PEP concentration increased, and V_Ppc _and V_PTS _increased for Pyk mutant as compared to the wild type strain, since PEP is the substrate for these pathway reactions. These were consistent with the previous experimental data [11,17-19]. The increase in Ppc flux caused OAA concentration to be increased. Then the reverse reaction of MDH became higher, resulting in the increase of MAL concentration. PYR was then backed up through Mez in Pyk mutant. Note that one mole of PYR is produced from one mole of glucose via PTS even for Pyk mutant. It was also observed that the increase in OAA concentration activated the Pck pathway. The increases in both Ppc and Pck fluxes yielded futile cycling. Overall, the result indicates the robustness feature of the cell metabolism as observed previously by experiments [11,17,18].

## Discussion

In the present study, we considered to simulate the main metabolism of *E. coli*. By considering the whole main metabolic pathways such as glycolysis, PP pathway and TCA cycle, we could estimate the specific ATP production rate, the specific NADPH production rate, and specific CO_2 _production rate. Those could be used to estimate the specific growth rate and the cell yield etc., and those were affected by the specific gene knockout. Thus, the simulation result is quite useful in identifying the mechanism of metabolic changes in terms of intracellular metabolite concentrations and the fluxes caused by the specific gene knockout. It should be noted that the flux balance analysis (FBA) has been known to be useful to predict the steady-state fluxes. However, since FBA is based on the mass balances with stoichiomatric matrix, it cannot treat the dynamic behavior. The effects of metabolite concentrations on the fluxes cannot be investigated by FBA, while it can be done by the kinetic equation-based approach. Moreover, although the metabolic flux analysis based on ^13^C-labeling experiments is powerful [12,13], it is method of analysis and cannot predict the flux changes caused by the change in culture environment and/or specific gene knockout.

It should be noted that the modeling and simulation of the whole central metabolic pathways are quite important, since the fluxes obtained allow us to estimate how ATP production rate and CO_2 _production rate etc. change with respect to time and/or the specific gene knockout, whereas part of the metabolic pathways such as only EMP pathway and/or the PP pathway cannot do this. In the present approach, since we considered the whole main metabolic pathways, the specific ATP production rate could be estimated for substrate level phosphorylation and oxidative phosphorylation with respect to time, and this could be utilized to estimate the cell growth rate. Moreover, the specific CO_2 _production rate could also be computed, and this can be used to estimate the yield.

Figure 2b indicates that the specific NADPH production rate, *v*_*NADPH *_is growth rate-dependent. It is usually difficult to predict the PP pathway fluxes using only enzymatic equations. Once we could estimate the cell growth rate based on ATP production rate (catabolism) as stated above, the PP flux may be estimated based on the relationship between the cell growth rate and NADPH production rate (anabolism) as given in Figure 2b. This reflected in the flux computations. Here we assumed that Entner Doudoroff pathway was inactive and the fluxes of G6PDH and 6PGDH were equal at steady state without loss of generality. Note that the computational algorithm for computing the flux through oxidative PP pathway requires repetitive evaluation as follows: Once the specific glucose consumption rate was given, all the intracellular fluxes can be computed using kinetic models for the main metabolic pathways. Then the specific ATP production rate was estimated by Eq.(3) with (4), and the specific growth rate was estimated by Eq.(5), using the relationship found from Figure 2a. The *v*_*NADPH *_was computed by Eq.(8) with the relationship of Figure 2b. The fluxes through G6PDH and 6PGDH were estimated from Eq.(7). Since the mass balance at G6P changes by the newly determined flux through G6PDH, the intracellular fluxes from G6P to Pgi were recalculated again and so on. Note that the fluxes of the oxidative PP pathways such as G6PDH, 6PGDH and/or ICDH were estimated, NADPH/NADP^+ ^concentrations can also be estimated if NADPH consumption rates at GDH (glutamate dehydrogenase) and other amino acids were taken into account.

As stated before, the predictability is one of the primary concerns for the simulation from the practical application point of view. Figure 5a and Additional file 4 indicate that Ppc knockout caused Pyk flux to be up-regulated and PEP concentration to be up-regulated. The increase of PEP concentration also caused GAP/DHAP concentration to be up-regulated. The Ppc knockout also caused OAA concentration to be significantly down regulated. Among the data investigated, only AcCoA concentration was different between the simulation and experimental results, where it became lower for the experiment, while it became higher for the simulation. This is due to the fact that AcCoA concentration increased by the increase in PDH flux, and the reduced flux of CS caused by low OAA concentration.

Different from Ppc knockout, Pck knockout resulted in significant reduction in the Ppc flux, and PEP concentration decreased in the simulation, as also shown by the experiment [15]. Moreover, the PEP, PYR, and AcCoA concentrations together with ICit concentration were down-regulated. This may be due to the reduction of AcCoA concentration which acts as an activator of Ppc [15]. Moreover, the glyoxylate pathway was activated in order to backup OAA.

As shown in Additional file 7, most of the glycolytic metabolites such as G6P/F6P, GAP/DHAP and PEP were higher for Pyk mutant, and the simulation result is consistent with the experimental data. The inhibition of Pfk by PEP is not so high, and the glycolysis fluxes became rather higher due to the increased PTS flux caused by the increase in PEP concentration. The increased flux of Ppc caused an increase in OAA and MAL concentrations. The concentration of PYR is supposed to be lower as observed in the experiment, but it shows the reverse result in the simulation. This may be due to the increase in PTS flux caused by the increase in PEP concentration. If we consider the mass balance for PYR, V_Pyk _is zero, V_PTS _increased, while V_Mez _and V_PDH _increased, and PYR concentration may decrease [11,17-19], while the simulation result shows reverse. The concentrations of all the TCA cycle metabolites such as ICIT, αKG, FUM, and SUC increased in the simulation, whereas the experimental data indicates the reverse trends. This may be due to the increase in PYR for the simulation. In consistency with the experimental data [11,17,18], the simulation result indicates that Pyk knockout has little effect on the cell growth characteristics (Figure 7a.). However, significant changes were observed in the metabolite concentrations and fluxes inside the cell, where the increased PEP concentration increased the flux of Ppc, which caused the increase of OAA and MAL concentrations and Mez flux to backup PYR. Those are consistent with enzyme activity data [17] and the flux result obtained by the ^13^C-labeling experiment.

In the present investigation, we focused on the regulation at the important branch point of PEP in view of energy generation and anapleorotic requirement. This may be extended to others mutants. However, the present model cannot simulate single gene knockouts for enzymes encoded by multiple genes, such as the operons *acnAB *encoding *Acn*, *sucAB*, *lpdA *encoding αKGDH, *sdhCDAB *encoding SDH, and *fumABC *encoding Fum in the TCA cycle. This is because, the present model does not include such gene level regulation.

## Conclusions

The simulation results indicate the usefulness in understanding the metabolic changes based on intracellular metabolite concentrations and fluxes, and how those affected the fermentation characteristics in response to specific pathway gene knockouts for both batch and continuous cultures. In conclusion, the present model could predict the metabolic changes due to these specific pathway knockout to some extent, and thus it is useful in understanding the metabolic regulation of the specific gene knockout mutant in practice.

## Methods

### Strains used and culture conditions

The strains used were *Escherichia coli *BW25113 (lacI^q ^rrn B_T14 _ΔlacZ_WJ16 _hsdR514 ΔaraBAD_AH22 _ΔrhaBAD_LD78_) and its *ppc *(JWK3928), *pck *(JWK3366) and *pykF *(JWK1666) mutants. The M9 synthetic medium was used for both batch and continuous cultivations, where it consists of 48 mM Na_2_HPO_4_, 22 mM KH_2_PO_4_, 10 mM NaCl, 40 mM (NH4)_z_SO_4 _and 4 g/l of glucose. The following components were filter-sterilized separatory and then added (per liter of final volume): 1 ml 1 M MgSO_4_, 1 ml vitamin B1 (1 mgl stock), 1 ml 0.1 mM CaCl_2_, and 10 ml trace element solution containing (per liter): 0.55g CaCl_2 _1g FeCl_3_, 0.1 mg/l MnCl_2_.4H_2_O, 0.17 g ZnCl_2_, 0.043 g CuCl_2_.2H_2_O, 0.06 g CoCl_2_.2H_2_O, and 0.06 g Na_2_MoO_4_.2H_2_O). Batch and continuous cultures were conducted at 37°C in a working volume of 1 l in a 2l fermentor (M-100, Tokyo Rikakikai, Japan) equipped with pH and temperature sensors. The air flow rate was maintained at 1 l min^-1 ^and 300-400 RPM, which ensured the aerobic condition where the dissolved oxygen concentration was kept at 3-4 ppm. The pH of the culture was controlled at 7.0 ± 0.1 by automatic addition of either 2.0 M HCl or 2.0 M NaOH with a pH controller. The CO_2 _and O_2 _concentrations in the off-gas were measured by an off-gas analyzer (DZX-2562, Able Co. Japan).

### Measurement for extracellular metabolites concentrations

The 5 ml of samples were taken from the culture broth and rapidly quenched into 15 ml of 60% (v/v) aqueous methanol containing 70 mM HEPES, where quenching solution was kept at -80°C before used. The cells were separated from the culture by centrifugation at 10,000 ×g for 15 min at 0°C. To extract intracellular metabolites from the cell pellet, 500 ml of 50% methanol was added, and the cells were re-suspended by vortexing the mixture. Then 2 ml of 35% of perchloric acid was added, which was pre-cooled on ice, and vortexed again for 10 s. After one freeze-thaw cycle, proteins and cell fragments were removed by centrifugation at 12,000 ×g for 30 min at 0°C. The clear suparnatant was neutralized by adding collected supernatant, and then 895 μm of 5 M K_2_KO_3 _was added. Afterward, precipitated perchlorates were removed by another centrifugation step (12,000 ×g at 0°C for 10 min), the clear supernatant was collected, and they were stored as 200 μm aliquots at -20°C for further analysis [20,21].

The intracellular metabolite concentrations were determined by enzymatic methods, where those were performed mainly as described by [22,23] and others [3,20,21,24] after some modifications. Enzymatic determinations of metabolites were performed in 50 mM tri ethanolamine buffer (TEA) at pH 7.6. For example, G6P concentration was determined in the presence of 5 mM MgSO4 and 0.48 mM NADP^+ ^after addition of 0.7 U ml^-1 ^of G6PDH. The measurements were made using Luminescence Spectrophotometer (LS55, Perkin Elmer, UK). The chemical determination was made at 37°C [20].

### 13C-labeling experiments and sample preparation

The biomass sample was kept on ice for 2-3 minutes, and the sample was centrifuged at 6,000 rpm at 2°C for 15 minutes [25]. The cell pellets were washed three times with 20 mM Tris-HCl at pH 7.6 and suspended in 10 ml of 6 M HCl. The mixture was then hydrolyzed at 105°C for 15 hours in a sealed glass tube. During acid hydrolysis, tryptophan and cysteine were oxidized, and glutamine and asparagine were deaminated. The hydrolysate was evaporated to dryness. The dried material was dissolved in milli-Q water and filtered through a 0.22 μm pore-size filter and evaporated to dryness. About 1.5 ml acetonitrile was added in the dried hydrolyte and incubated at room temperature overnight. After the color of liquid turned a color of pale yellow, it was filtrated through 0.22 μm pore-size filter. The filtrate was then derivatized by N-(tert-butyldimethylsilyl)-N-methyl-trifluoroacetamide (MTBST-FA) (Aldrich, USA) at 110C for 30 minutes and was transferred to GC-MS sample tube for analysis [25].

^13^C-labeling experiments were initiated after the culture reached steady state, which was inferred from the stable oxygen and carbon dioxide concentrations in the off-gas and stable OD in the effluent medium for at least twice as long as the residence time. The feed medium with 4 g/l of unlabeled glucose was then replaced by an identical medium containing 0.4 g of [U-^13^C] glucose, 0.4 g of [1-^13^C] glucose, and 3.2 g of naturally labeled glucose per liter. Biomass samples for GC-MS analysis were taken after one residence time. Sample preparation, analytical procedures for GC-MS analysis, and flux computation are given elsewhere [16-18,25].

### Metabolic flux analysis

Preparation of biomass hydrolysates and recording of the GC-MS spectra (PerklinElmer, Germany) were made as described previously [25-27]. The program Turbomass Gold (Perklin Elmer,Germany) was used for peak assignment and MS data processing. The main idea is to perform isotopomer balance on carbon atoms in order to track the fate of the labelled carbon atoms from the substrate [28]. Isotopomer balance enables us to determine the isotopomer distributions of the intracellular metabolites in the central metabolic network. Since the isotopomer distribution of amino acids can be inferred from the isotopomer distributions of their corresponding precursors, the GC-MS signals for the amino acids can then be simulated. A set of flux distributions is then determined by minimizing the differences between the experimental and simulated data. For the estimation of GC-MS signals using isotopomer balance, three types of corrections were made to take into account the effects of natural abundance, non-steady state condition, and skewing effect. First, the isotopomer distributions of the input substrate were corrected for natural abundance in the unlabeled glucose and impurity in the labeled glucose. Second, the isotopic steady state condition is normally not attained at the time of harvesting biomass. Thus, the simulated data have to be corrected based on the actual harvesting time by assuming first order washout dynamics [29]. Finally, the simulated GC-MS data were corrected for the natural isotope abundance of O, N, H, Si, S, and C atoms in the derivatizing agent using the correction matrices [30].

### Dynamic equations

Referring to Figure 1, the dynamic equations may be described based on mass balances as

(1a) $$\frac{d[X]}{dt}=\mu [X]$$

(1b) $$\frac{d[GLC^{ex}]}{dt}=-v_{PTS}[X]$$

(1c) $$\frac{d[G6P]}{dt}=v_{PT\text{S}}-v_{PGI}-v_{G6PDH}-\mu [G6P]$$

(1d) $$\frac{d[F6P]}{dt}=v_{PGI}-v_{PFK}+v_{TKTB}+v_{TAL}-\mu [F6P]$$

(1e) $$\frac{d[FDP]}{dt}=v_{PFK}-v_{ALDO}-\mu [FDP]$$

(1f) $$\frac{d[G\text{A}P]}{dt}=2v_{ALDO}-v_{GAPDH}+v_{TKTA}+v_{TKTB}-v_{TAL}-\mu [G\text{A}P]$$

(1g) $$\frac{d[PEP]}{dt}=v_{GAPDH}-+v_{PCK}-v_{PTS}-v_{PYK}-v_{PPC}-\mu [PEP]$$

(1h) $$\frac{d[PYR]}{dt}=v_{PYK}+v_{PTS}+v_{MEZ}-v_{PDH}-\mu [PYR]$$

(1i) $$\frac{d[AcCoA]}{dt}=v_{PDH}+v_{ACS}-v_{CS}-v_{PTA}-\mu [AcCoA]$$

(1j) $$\frac{d[ICIT]}{dt}=v_{CS}-v_{ICDH}-v_{ICL}-\mu [ICIT]$$

(1k) $$\frac{d[2KG]}{dt}=v_{ICDH}-v_{2KGDH}-\mu [2KG]$$

(1l) $$\frac{d[SUC]}{dt}=v_{2KGDH}+v_{ICL}-v_{SDH}-\mu [SUC]$$

(1m) $$\frac{d[FUM]}{dt}=v_{SDH}-v_{FUM}-\mu [FUM]$$

(1n) $$\frac{d[MAL]}{dt}=v_{FUM}+v_{MS}-v_{MDH}-v_{MEZ}-\mu [MAL]$$

(1o) $$\frac{d[OAA]}{dt}=v_{MDH}+v_{PPC}-v_{CS}-v_{PCK}-\mu [OAA]$$

(1p) $$\frac{d[GOX]}{dt}=v_{ICL}-v_{MS}-\mu [GOX]$$

(1q) $$\frac{d[ACP]}{dt}=v_{PTA}-v_{ACK}-\mu [ACP]$$

(1r) $$\frac{d[ACE^{ex}]}{dt}=(v_{ACK}-v_{ACS})\left[X\right]$$

(1s) $$\frac{d[6PG]}{dt}=v_{G6PDH}-v_{6PGDH}-\mu [6PG]$$

(1t) $$\frac{d[Ru5P]}{dt}=v_{6PGDH}-v_{RPE}-v_{RPI}-\mu [Ru5P]$$

(1u) $$\frac{d[R5P]}{dt}=v_{RPI}-v_{TKTA}-\mu [R5P]$$

(1v) $$\frac{d[X5P]}{dt}=v_{RPE}-v_{TKTA}-v_{TKTB}-\mu [X5P]$$

(1w) $$\frac{d[S7P]}{dt}=v_{TKTA}-v_{TAL}-\mu [S7P]$$

(1x) $$\frac{d[E4P]}{dt}=v_{TAL}-v_{TKTB}-\mu [E4P]$$

where [·] denotes the concentration, and the abbreviations for the metabolites are explained in Nomenclature. *μ *is the specific growth rate and *v*_*i *_are the intracellular fluxes. The superscript "^ex^" means extra cellular. Note that the last term with *μ *in each equation denotes the dilution effect due to the increase in cell volume as the cell grows [8]. As shown in Figure 1, GAP and DHAP were lumped together. A sequence of enzymatic reactions by GAPDH, Pgk, Pgm and Eno can be considered to be in equilibrium, and thus it was assumed to be one lumped reaction from GAP to PEP, for simplicity. The overall simulation procedure is given in Additional file 8.

### Modeling for the cell growth rate

The estimation for the cell growth rate is by far important. In particular, it is critical for the prediction of the specific gene knockout mutant. The most typical equation for the cell growth rate is the Monod equation:

(2a) $$\mu (S)=\;\frac{\mu_{m}S}{K_{S}+S}$$

where *S *is the substrate concentration and *S *≡ [*Glc^ex ^*] in the present case. The drawback of this equation is that the cell will keep growing at *μ*_*m *_as far as *S *≫ *K*_*s *_where *K*_*s *_is usually quite small in the case of the utilization of glucose as the substrate. This does not reflect the fermentation characteristics in practice. Therefore, we introduced another term such as

(2b) $$\mu (S,X)=\mu_{m}{\left(1-\frac{X}{X_{m}}\right)}^{n}\frac{S}{K_{S}+S}$$

where *X *is the cell concentration and *X*_*m *_is the final value of *X *in the batch culture, and n is the model parameter. *X*_*m *_may be set as *S*(0)*Y*_*S/X *_where *S *(0) is the initial substrate concentration, and *Y*_*S/X *_is the yield coefficient. Although this equation can be used to simulate the dynamic behavior in batch culture, Eq. (2b) may not be able express the cell growth rate for the mutants. We, therefore, introduce another idea by taking into account the effect of ATP generation on the cell growth rate based on experimental observation.

The ATP production is made either by substrate level phosphorylation and oxidative phosphorylation, where the reducing power of NADH and FADH_2 _can contribute in generating ATP via oxidative phosphorylation. The pathways involved in electron transfer and oxidative phosphorylation have variable stoichiometry due to the use of different dehydrogenases and cytochromes. Namely, the NADH dehydrogenase NDH-I encoded by *nuo *gene transports 2 H^+^/e^-^, while NDH-II encoded by *ndh *gene transports 0 H^+^/e^- ^[31]. Quinol then oxidases cytochromes cyt bO_3 _and cyt bd to transport 2 H^+^/e^- ^and 1 H^+^/e^- ^, respectively [31]. The number of H^+ ^transported into the cell by the membrane bound H^+^-ATPase to phosphorylate ADP to form ATP has been estimated to be 2-4, with 3 being most likely under aerobic condition [32]. The P/O ratio is a non integer value in practice. Here we consider P/O ratio to be the model parameter to be tuned by fitting the experimental data. Thus the specific ATP production rate may be estimated by the following equation:

(3) $$v_{ATP}=OP_{NADH}+OP_{FADH_{2}}+v_{GAPDH}+v_{Pyk}+v_{Ack}+v_{SDH}-v_{Pfk}-v_{Pck}$$

(3)

Where *OP*_*NADH *_and $OP_{FADH_{2}}$ is the specific ATP production rates via oxidative phosphorylation, which may be estimated by

(4a) $$OP_{NADH}=(v_{GAPDH}+v_{PDH}+v_{ICDH}+v_{2KGDH}+v_{MDH})\times (P/O)$$

and

(4b) $$OP_{FADH_{2}}=v_{SDH}\times (P/O)'$$

where: (*P*/*O*) and (*P*/*O*)' are the P/O ratios for NADH and FADH_2_, respectively.

Figure 2a implies the linear relationship between the specific growth rate and the specific ATP production rate, and then Eq (2b) may be further modified as

(5) $$\mu (S,X,v_{ATP})=\mu_{m}{\left(1-\frac{X}{X_{m}}\right)}^{n}\frac{S}{K_{s}+S}k_{ATP}v_{ATP}(•)$$

Where *v*_*ATP *_(•) is the specific ATP production rate computed from the fluxes, and *v*_*ATP *_is the adjustable model parameter.

### Kinetic equations for the enzyme kinetics

The kinetic equations for the various enzyme-catalyzed reactions considered in the present study are given in Table 1. The specific growth rate was assumed to be expressed by Eq.(1), where Eq.(1a) in Table 1 was used for the cell growth phase as state above, while Eq.(1b) was used for the late growth phase where acetate was used as a carbon source.

The glucose transport via phosphotransferase system (PTS) has been extensively investigated and is catalyzed by a sequence of enzymes such as EI, HPr, EIIA^Glc ^and EIICB^Glc^. These were encoded by such genes as *ptsHI*, *crr *and *ptsG*. It may be assumed that EIICB^Glc ^can be either phosphorylated first or bind with glucose first, although the phosphorylation of EIIB may be facilitated by binding EIIC to glucose. Based on these assumptions, the kinetic rate equation may be derived for the glucose transport through PTS as given by Eq.(2) in Table 1[33], where Glc^ex ^and PEP are the substrates for the reaction, and PYR and G6P are the products, which inhibit the reaction rate as those are accumulated.

The phophoglucose isomerase (Pgi) is the first enzyme in glycolysis, and the two Pgi isoenzymes have been lumped into one rate equation [34]. The phosphofructokinase (Pfk) is encoded by *pfkA *and *pfkB *in *E. coli*, where Pfk-1 encoded by *pfkA *is dominant, accounting for 90% of the total activity [35]. Here, we, therefore, considered only Pfk-1, where it is inhibited by PEP [36], and its reaction rate may be expressed as given by Eq.(4) of Table 1, where F6P is the substrate for this reaction, and PEP inhibits *v*_Pfk _as it accumulates [8]. For FDP aldolase reaction, Eq.(5) of Table 1 was considered [8], where FDP is the substrate and GAP is the product. The minus term in the numerator is for the backward reaction. For GAPDH reaction, Eq.(6) of Table 1[8] was considered, where GAP is the substrate, and PEP is the product for this reaction. Note that the reactions from GAP to PEP were lumped into GAPDH reaction for simplicity. This reaction rate was assumed to be inhibited by NADH as [NADH]/[NAD] ratio increases. The pyruvate kinase (Pyk) reaction is catalyzed by two isoenzymes, PykI and PykII, encoded by *pykF *and *pykA*, respectively. PykI is activated by FDP and inhibited by ATP, whereas PykII is activated by AMP. Here we lumped these together, and the rate equations may be expressed as given by Eq.(7) of Table 1, where PEP and ADP are the substrates for this reaction and inhibited by ATP [8]. Moreover, Eq.(7) indicates that FDP and AMP are the activators of the reaction.

It is quite important to correctly simulate the fluxes at the branch point of PEP, where the enzyme activities of Pyk and Ppc determine the fluxes. Based on the experimental data [37], it is known that the reaction catalyzed by Ppc exhibits a hyperbolic function with respect to PEP concentration, and the reaction rate is usually low without any activator, where AcCoA is a very potent activator, and FDP alone exhibits no activation, but it produces a strong synergistic activation with AcCoA. Based on these observations, Eq.(8) of Table 1 may be considered [38]. Consider now the reaction in the reverse direction, namely Pck. The kinetics of the Pck in *E. coli *may be assumed to follow the rapid equilibrium mechanism [39]. Thus, we may express the rate equation as given by Eq.(9) of Table 1, where OAA and ATP are the substrates for this reaction, and the reaction rate is inhibited by its products such as PEP and ADP [15].

For the rate equation of PDHc, the hyperbolic function (Michaelis-Menten type) was considered with respect to PYR, and it was assumed to be inhibited by NADH as [NADH]/[NAD] ratio increases as given by Eq.(10) of Table 1[40]. The acetate formation is of important concern in practice for the cell growth and the specific metabolite production in *E. coli*. The cells undergo a metabolism switch associated with the production and utilization of acetate. When enough glucose is present around the cell, the cell produces and excretes acetate. Once glucose is consumed or becomes low level, the cells utilize acetate instead of excreting it. This acetate-associated metabolic switch occurs just as the cells begin to decelerate growth, or fall into stationary phase in the batch culture. The first pathway for acetate production is Pta-Ack pathway, where the reaction catalyzed by Pta and Ack proceeds through an unstable, high energy, acetyl phosphate (AceP) intermediate. For the Pta reaction, Eq.(11) of Table 1 was considered [40,41]. The rate equation for Ack was considered as expressed by Eq.(12) of Table 1[40]. During the stationary phase when glucose was depleted, the cells scavenge for the acetate by using the 2^nd ^pathway of Acs. This reaction catalyzed by Acs proceeds through an enzyme-bound acetyladenylate (Acetyl-AMP) intermediate. Acs has high affinity to acetate while Ack-Pta has low affinity. The rate equation for Acs was assumed to be expressed by the simple Michaelis-Menten equation as given by Eq.(13) of Table 1[42].

For citrate synthase reaction, Eq.(14) in Table 1[43] was considered, where AcCoA and OAA are the substrates, and the reaction is inhibited by NADH. Another important branch point is at isocitrate (ICIT) branch point in the TCA cycle, where ICDH (high affinity to ICIT, K_m _= 8 mM) and Icl (low affinity to ICIT, K_m _= 406 mM) compete for ICIT. This branch point is under both gene level regulation by *aceBAK *operon and the enzyme level regulation by the phosphorylation/dephosphorylation of ICDH. Here, we considered the rate equation for ICDH as given by Eq.(15) of Table 1[44], where ICIT is the substrate, and the reaction is inhibited by NAD(P)H as [NAD(P)H]/[NAD(P)] ratio increases and as [αKG] increases. For KGDH reaction, Eq.(18) of Table 1 was considered, where αKG and CoA are the substrates, and the reaction is assumed to be inhibited by NADH as [NADH]/[NAD] ratio increases [45]. For SDH reaction, Eq.(19) of Table 1 was considered, where SUC is the substrate and FUM is the product [45]. For fumarase reaction, Eq.(20) of Table 1 was considered, where FUM is the substrate, and the reaction is inhibited by its product MAL [45]. For MDH reaction, Eq.(21) of Table 1 was considered [45], where MAL is the substrate, and the reaction is inhibited by its products OAA and NADH as these concentrations increase. For malic enzyme reaction, Eq.(22) of Table 1 was considered [45].

As for the glyoxylate pathway, Eq. (16) of Table 1 was considered for Icl, ICIT being the substrate of reaction, inhibited by high accumulation of its products SUC and GOX [46]. For malate synthase reaction, Eq.(17) of Table 1 was considered, where GOX and AcCoA are the substrates, and the reaction is inhibited by its product MAL [46].

For G6PDH and 6PGDH reactions in the PP pathway, Eqs.(23) and (24) of Table 1 were considered, where G6P is the substrate of the former reaction inhibited by NADPH, and 6PG is the substrate of the latter reaction inhibited by NADPH [8]. Other equations for PP pathway such as Rpe, Rpi, TktA, TktB, and Tal were considered in the form of mass-action kinetic, given in Eqs. (25)-(29) in Table 1[8].

Once the fluxes were determined, the specific CO_2 _production rate $v_{CO_{\text{2}}}$ was assumed to be estimated by

(6) $$v_{CO_{2}}=v_{PDH}+v_{Pck}+v_{ICDH}+v_{KGDH}+v_{Mez}+v_{6PGDH}-v_{Ppc}$$

and the total CO_2 _production rate was computed by the product of net production of CO_2 _and biomass concentration, i.e. $v_{CO_{\text{2}}}$ [X].

Moreover, the specific NADPH production rate *v*_*NADPH *_may also be estimated by

(7) $$v_{NADPH}=v_{G6PDH}+v_{6PGDH}+v_{MEZ}+(v_{ICDH})$$

It should be noted that the flux determination in the PP pathway is not easy for the enzyme-based kinetic equations. In particular, it is not easy to accurately predict the flux of G6PDH at the branch point of G6P. Figure 2b shows the relationship between the specific growth rate *μ *and *v*_*NADPH *_computed based on the flux estimates obtained from ^13^C-labeling experiments [14,15], indicating the linear relationship between the two as:

(8) $$v_{NADPH}=k_{NADPH}\mu$$

Once we identified *k*_*NADPH *_value from Figure 2b, we may compute *k*_*NADPH *_after *μ *was computed. Then, the flux of G6PDH may be estimated.

As stated in introduction, it is not easy, even if it is not impossible, to express gene-level regulation by mathematical equations. Here, we alternatively introduced several rules for the simulation based on the experimental observations and the roles of global regulators such as given in Additional file 3: Table S1, where the rules 1-3 in Additional file 3: Table S1 came from the experimental observations, while Rule 4 and Rule 5 are known by gene level regulations [47,48]. Rule 6 came from our previous analysis [11].

### Nomenclature

Metabolites

2KG: 2-Keto-D-gluconate; 6PG: 6-Phosphogluconolactone; ACE: Acetate; AcP: Acetyl phosphate; AcCoA: Acetyl-CoA; ASP: Aspartate; ADP: Adenosine diphosphate; ATP: Adenosine-5'-triphosphate; AMP: Adenosine monophosphate; DHAP: Dihydroxyacetone phosphate; E4P: Erythrose 4-phosphate; F6P: Fructose 6-phosphate; FDP: Fructose 1,6-bisphosphate; FUM: Fumarate; G6P: Glucose-6-phosphate; GAP: Glyceraldehyde 3-phosphate; ICIT: Isocitrate; NAD/NADH: Nicotinamide adenine dinucleotide; NADP/NADPH: Nicotinamide adenine dinucleotide phosphate; MAL: Malate; OAA: Oxaloacetate; P: Phosphate; PEP: Phosphoenolpyruvate; PYR: Pyruvate; R5P: Ribulose 5-phosphate; Ru5P: Ribose 5-phosphate; S7P: Sedoheptulose 7-phosphate; SUC: Succinate; X5P: Xylulose 5-phosphate

Protien (enzyme)

2KGDH: 2-Keto-D-gluconate Dehydrogenase; Ack: Acetate kinase; Acs: Acetyl coenzyme A synthetase; AcP: Acetyl phosphate; Aldo: Aldolase; CS: Citrate synthase; EI: Cytoplasmatic protein (enzyme1); EII, EIIB, EIIC,: Carbohydrate specific (enzyme II); EIIA_Glc_,/EIICB_Glc_: Enzyme for glucose; Eno: Enolase; Fum: Fumarase; G6PDH: Glucose-6-phosphate dehydrogenase; GAPDH: Glyceraldehyde 3-phosphate dehydrogenase; ICDH: Isocitrate dehydrogenase; HPr: Histidine containing protien; Icl: Isocitrate lyase; MDH: Malate dehydrogenase; Mez: Malic enzyme; Ms: Malate synthase; Pck: Phosphoenolpyruvate carboxykinase; PDH: Pyruvate dehydrogenase; Pfk/Pfk-1: Phosphofructokinase-1; Pgi: Phosphoglucose isomerase/Glucosephosphate isomerase; Pgk: Phosphoglycerate kinase; Pgm: Phosphoglycerate mutase; Ppc: PEP carboxylase; Pps: Phosphoenolpyruvate synthase; Pta: Phosphotransacetylase; PTS: Phosphotransferase system; Pyk: Pyruvate kinase; Rpe: Ribulose phosphate 3-epimerase; Rpi: Ribulose 5-phosphate 3-isomerase; SDH: Succinate dehydrogenase; Tal: Transaldolase; TktA: TransketolaseI; TktB: TransketolaseII

Gene

*aceBAK*: operon which encodes the metabolic and regulatory enzymes of the glyoxylate bypass; *cra*: Catabolite repressor/activator; *crr*: Catabolite repressor; *fadR*: Fatty acid metabolism regulator; *icdA*: Isocitrate dehydrogenase A; *iclR*: Transcriptional repressor IclR; *pckA*: Phosphoenolpyruvate carboxykinase gene; *pfkA, pfkB*: Phosphofructokinase gene; *ppsA*: Phosphoenolpyruvate synthase gene; *ptsG, ptsHI*: Pts gene; *pykA, pykF*: Pyruvate kinase gene

## Competing interests

The authors declare that they have no competing interests.

## Authors' contributions

TAAK made modeling, experiment and computer simulation, AAM made some TCA cycle modeling and simulation, AMK supervised computer simulation, JM made formulation, KS made modeling and manuscripts preparation. All authors read and approved the final manuscripts.

## Supplementary Material

Additional file 1 **Effects of initial metabolite concentrations on the fermentation characteristics**. Click here for file

Additional file 2 **Simulation result of wild type and Ppc mutant in batch culture**. Click here for file

Additional file 3 **Table S1 - Ruled used for the simulation**. Click here for file

Additional file 4 **Simulation result of wild type and Ppc mutant in continuous culture**. Click here for file

Additional file 5 **Simulation result of wild type and Pck mutant in continuous culture**. Click here for file

Additional file 6 **Simulation result of wild type and Pyk mutant in batch culture**. Click here for file

Additional file 7 **Simulation result of wild type and Pyk mutant in continuous culture**. Click here for file

Additional file 8 **Computational procedure for the simulation of fitting experimental data**. Click here for file
