# Supplementary material for: Modeling and simulation of the main metabolism in Escherichia coli and its several single-gene knockout mutants with experimental verification
Source: Microb Cell Fact. 2010 Nov 19;9:88. doi: 10.1186/1475-2859-9-88 (PMC2999585; doi:10.1186/1475-2859-9-88)
Supplement: Additional file 1 — Effects of initial metabolite concentrations on the fermentation characteristics. [file 1475-2859-9-88-S1.PDF]

Additional file 1: Effects of initial metabolite concentrations on the fermentation chacacteristics

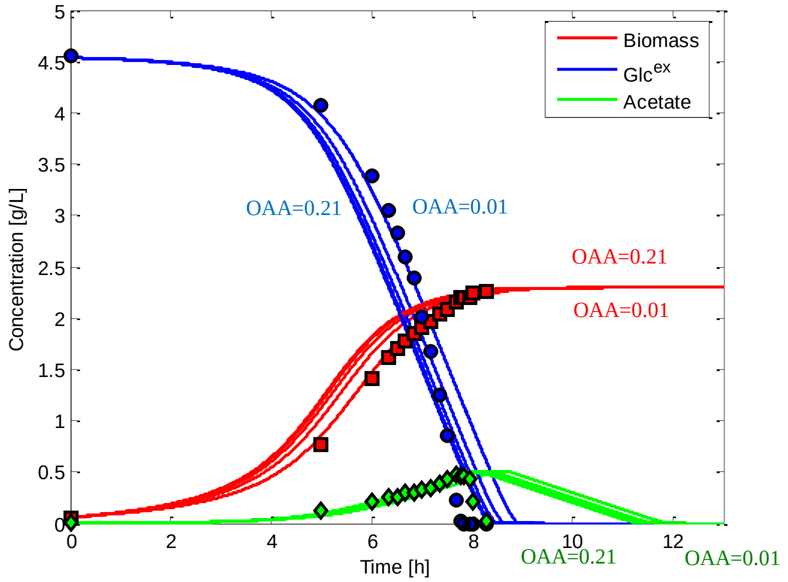

(a) Effect of OAA concentrations (0.01, 0.016, 0.011, 0.16, 0.21).

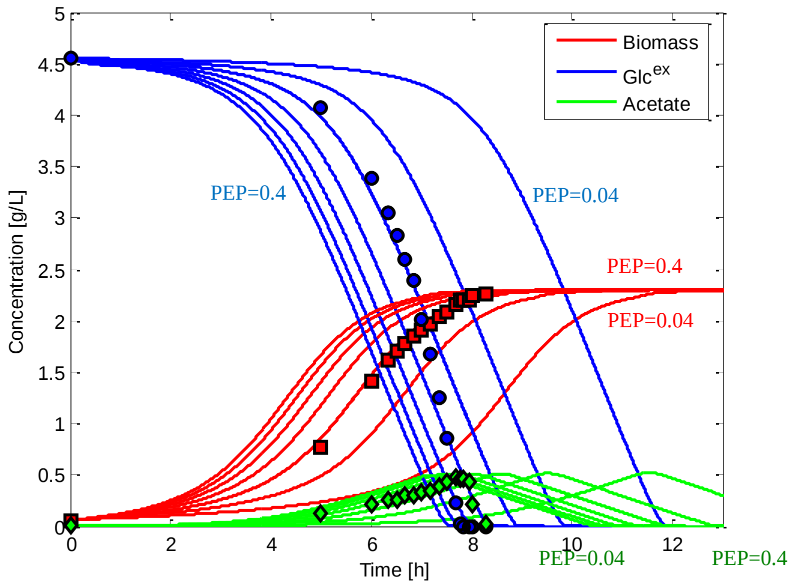

(b) Effect of PEP concentrations (0.04, 0.1, 0.16, 0.22, 0.28, 0.34, 0.4).
