# Supplementary material for: Modeling and simulation of the main metabolism in Escherichia coli and its several single-gene knockout mutants with experimental verification
Source: Microb Cell Fact. 2010 Nov 19;9:88. doi: 10.1186/1475-2859-9-88 (PMC2999585; doi:10.1186/1475-2859-9-88)
Supplement: Additional file 2 — Simulation result of wild type and Ppc mutant in batch culture. [file 1475-2859-9-88-S2.PDF]

Additional file 2: Simulation result of wild type and Ppc mutant in batch culture.

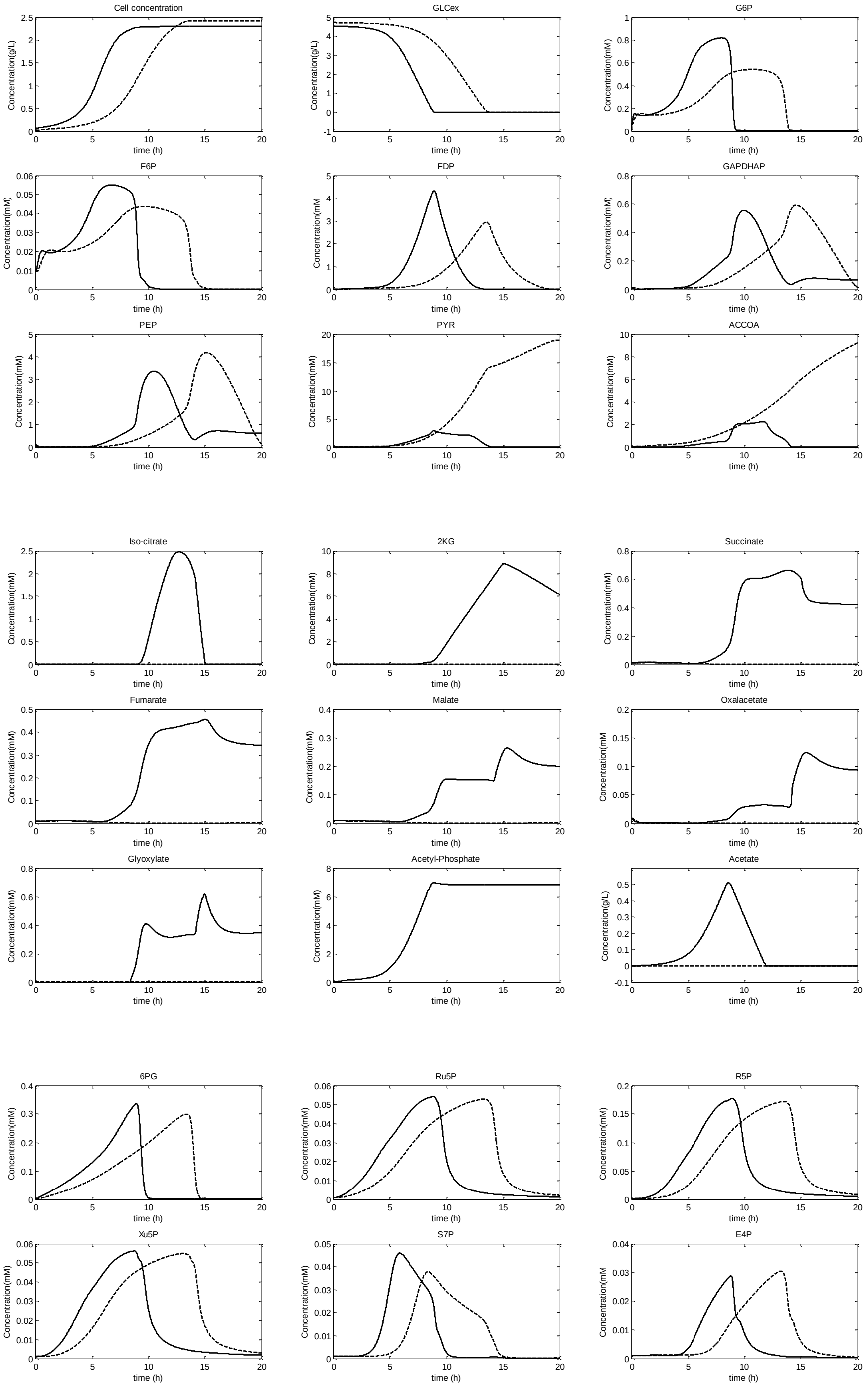

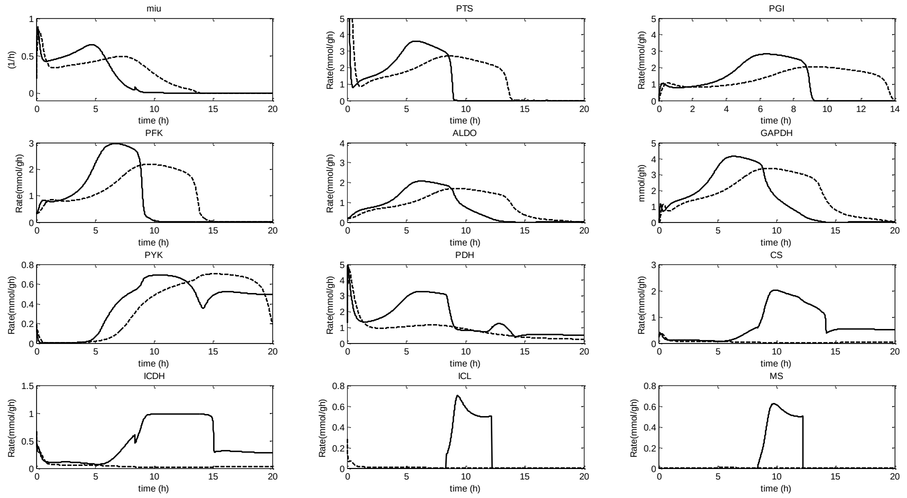

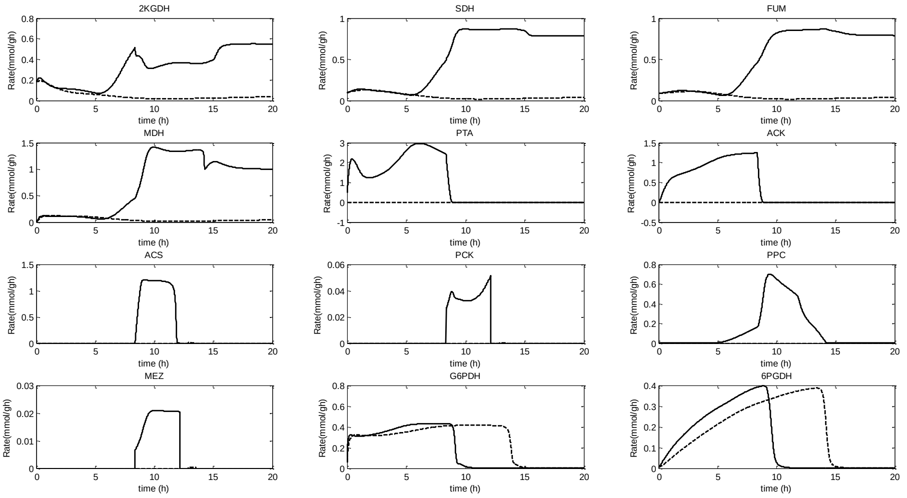

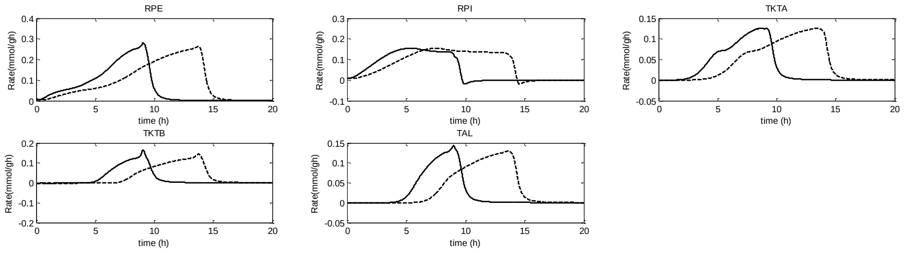

Note: The solid line represent wild type and dotted line represent the mutant.
