# Supplementary material for: Modeling and simulation of the main metabolism in Escherichia coli and its several single-gene knockout mutants with experimental verification
Source: Microb Cell Fact. 2010 Nov 19;9:88. doi: 10.1186/1475-2859-9-88 (PMC2999585; doi:10.1186/1475-2859-9-88)
Supplement: Additional file 3 — Table S1 - Ruled used for the simulation. [file 1475-2859-9-88-S3.PDF]

Additional file 3: Table S1: Ruled used for the simulation

Rule 1:
IF glucose concentration was present (more than 1g/l)
THEN glyoxylate pathway is repressed

Rule 2:
IF glucose was present (more than 1g/l)
THEN Pta-Ack pathway is active, while Acs and Mez are inactive

Rule 3:
IF glucose was less than 1 g/l, and acetate was present
THEN Acs is active in such a way that $v_{\max}^{Acs} = v_{\max}^{Acs}(1-[Glc])$, and *cra* gene is activated, and *fadR* and *iclR* genes are repressed

Rule 4:
IF *cra* gene was activated,
THEN *aceA*(Icl),*aceB*(MS),*icdA*(ICDH),*maeB*(Mez), *ppsA*(Pps), *pckA*(Pck) are activated, whereas *pykF*(Pyk), *pfkA*(Pfk), *ptsHI*(PTS) are repressed

Rule 5:
IF *fadR* and *iclR* were repressed,
THEN *aceBAK* operon is activated, and thus the glyoxylate pathway becomes active

Rule 6:
IF OAA concentration became low,
THEN the anaplerotic reaction such as glyoxylate pathway becomes active
