# Supplementary figures and images for: Modeling and simulation of the main metabolism in Escherichia coli and its several single-gene knockout mutants with experimental verification
Source: Microb Cell Fact. 2010 Nov 19;9:88. doi: 10.1186/1475-2859-9-88 (PMC2999585; doi:10.1186/1475-2859-9-88)

**Additional file 8:** Computational procedure for the simulation of fitting experimental data

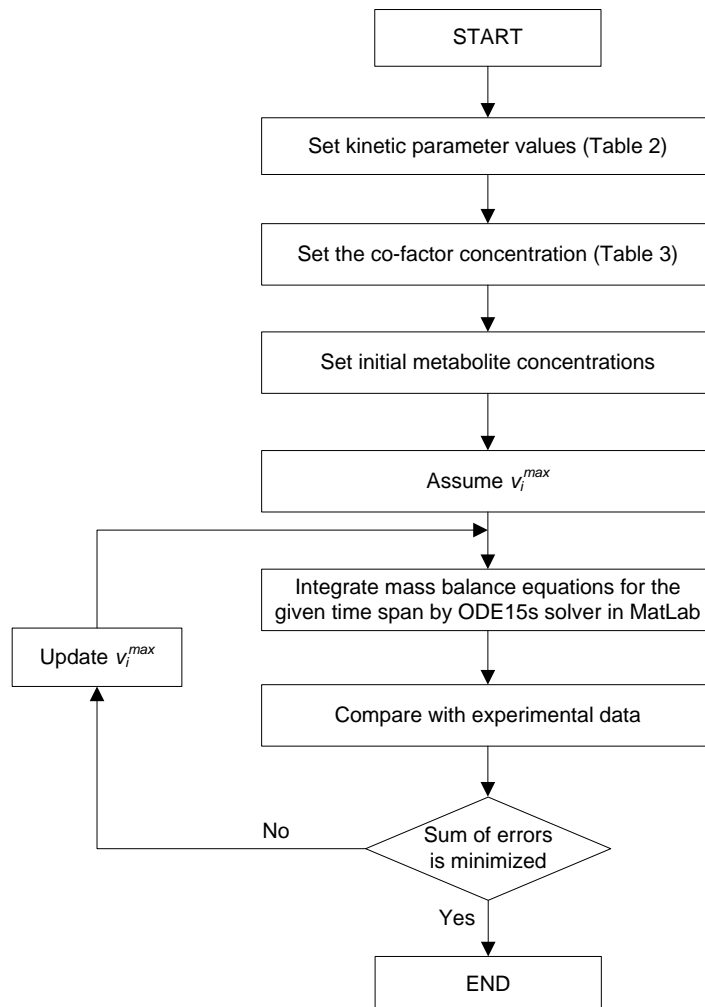

Supplement: Additional file 8 — Computational procedure for the simulation of fitting experimental data. [file 1475-2859-9-88-S8.PDF]
